# Chemical and Biological Study of Novel Aplysiatoxin Derivatives from the Marine Cyanobacterium *Lyngbya* sp.

**DOI:** 10.3390/toxins12110733

**Published:** 2020-11-23

**Authors:** Hui-Hui Zhang, Xin-Kai Zhang, Ran-Ran Si, Si-Cheng Shen, Ting-Ting Liang, Ting-Ting Fan, Wei Chen, Lian-Hua Xu, Bing-Nan Han

**Affiliations:** 1 Department of Development Technology of Marine Resources, College of Life Sciences and Medicine, Zhejiang Sci-Tech University, Hangzhou 310018, China; huihuizhang09@163.com (H.-H.Z.); xinkaiz2020@163.com (X.-K.Z.); ammashen7@163.com (S.-C.S.); mmftt1994@163.com (T.-T.F.); weichen120903@163.com (W.C.); 2 School of Materials Science and Engineering, Zhejiang Sci-Tech University, Hangzhou 310018, China; Siranran95@163.com; 3 School of Chemical and Environmental Engineering, Shanghai Institute of Technology, Shanghai 201418, China; liang_tting@163.com

**Keywords:** marine cyanobacterium, *Lyngbya* sp., aplysiatoxin, Kv1.5 inhibitory activity, brine shrimp toxicity

## Abstract

Since 1970s, aplysiatoxins (ATXs), a class of biologically active dermatoxins, were identified from the marine mollusk *Stylocheilus longicauda*, whilst further research indicated that ATXs were originally metabolized by cyanobacteria. So far, there have been 45 aplysiatoxin derivatives discovered from marine cyanobacteria with various geographies. Recently, we isolated two neo-debromoaplysiatoxins, neo-debromoaplysiatoxin G (**1**) and neo-debromoaplysiatoxin H (**2**) from the cyanobacterium *Lyngbya* sp. collected from the South China Sea. The freeze-dried cyanobacterium was extracted with liquid–liquid extraction of organic solvents, and then was subjected to multiple chromatographies to yield neo-debromoaplysiatoxin G (**1**) (3.6 mg) and neo-debromoaplysiatoxin H (**2**) (4.3 mg). They were elucidated with spectroscopic methods. Moreover, the brine shrimp toxicity of the aplysiatoxin derivatives representing differential structural classifications indicated that the debromoaplysiatoxin was the most toxic compound (half inhibitory concentration (IC_50_) value = 0.34 ± 0.036 µM). While neo-aplysiatoxins (neo-ATXs) did not exhibit apparent brine shrimp toxicity, but showed potent blocking action against potassium channel Kv1.5, likewise, compounds **1** and **2** with IC_50_ values of 1.79 ± 0.22 µM and 1.46 ± 0.14 µM, respectively. Therefore, much of the current knowledge suggests the ATXs with different structure modifications may modulate multiple cellular signaling processes in animal systems leading to the harmful effects on public health.

## 1. Introduction

Marine cyanobacteria, known as blue-green algae, can yield cyanotoxins with many biologically active metabolites, which contained three major groups based largely on their primary toxicological effects: hepatotoxins, neurotoxins, and contact irritants [1,2]. Among them, aplysiatoxins (ATXs), a kind of dermatoxins, which belonged to contact irritants in cyanotoxins, had attracted extensive attentions due to its series of poisoning and allergic events to public health [2,3,4,5,6]. ATXs were first discovered from the digestive gland of the *Stylocheilus longicauda* in 1970s [7,8]. Moreover, further study revealed that these toxins produced from cyanobacteria, which are “prey” of the sea hares [9,10]. Subsequently, ATXs and their related analogues were isolated from *Oscillatoria nigroviridis*, *Schizothrix calcicola*, and *Moorea producens* (formerly *Lyngbya majuscule*), which all belonged to cyanobacterial species [11,12,13,14]. In the 1980s, ATXs were confirmed as the factor causing marine dermatitis, because the blooms of *Lyngbya majuscula* broke out on the beach of Oahu Island, Hawaii, with itching or burning, evolving into a blistering eruption [3]. In the 1990s, ATXs and its related compounds led to the causative agents of successive food poisoning cases, with diarrhea, vomiting, and a burning sensation in the throat and mouth [4,5,6].

**Figure 1:**
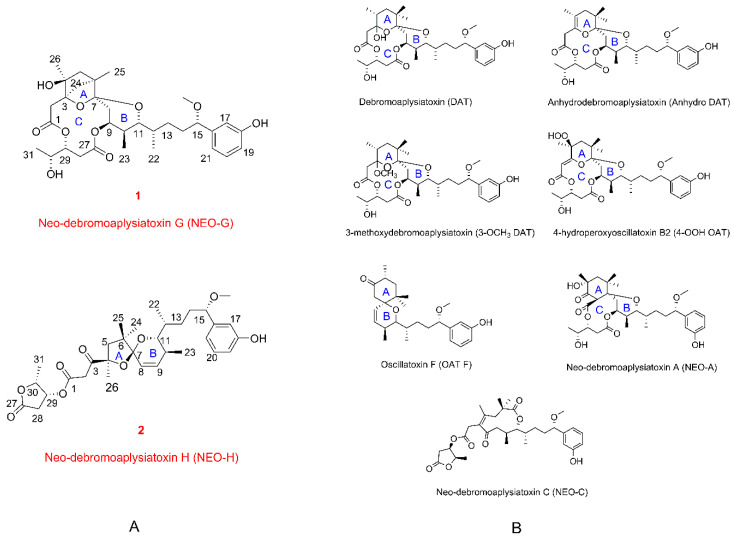
(**A**) Structures of neo-debromoaplysiatoxin G (**1**) and neo-debromoaplysiatoxin H (**2**); (**B**) structures of reported aplysiatoxin derivatives.

ATXs are a class of biologically active dermatoxins with tumor-promoting properties, anti-proliferative activity, antiviral activity, antileukemia activity, and pro-inflammatory actions [11,15,16,17,18]. So far, about 45 ATXs were identified from marine cyanobacteria, and mainly existed in *Lyngbya* [19,20,21,22,23,24]. Thus, the blooms of *Lyngbya* were often concerned with negative impacts on human health and economic implications [25]. In 2018, it was firstly reported that some ATXs exhibit potent blocking action against potassium channel Kv1.5 [20]. The potassium channel Kv1.5 expressed in cardiomyocytes of mammals [26,27], may be one of the key targets resulting in the harmful effects on public health. Based on this, our continuous study aimed to deepen the understanding of ATXs with different structural modifications that may modulate multiple cellular signaling processes in animal systems. In this study, we reported the extraction, structure elucidation, and biological activities of two neo-debromoaplysiatoxins (NEOs) featuring novel structural skeletons, neo-debromoaplysiatoxin G (**1**) and neo-debromoaplysiatoxin H (**2**), characterized by a bicyclo[2.1.1]tetrahydropyran and unique 5/6 fused-ring systems, respectively (Figure 1A).

## 2. Results

The freeze-dried sample of the cyanobacterium was extracted with liquid–liquid extraction of organic solvents. The resultant extracts were subjected to multiple chromatographies to yield neo-debromoaplysiatoxin G (**1**) (3.6 mg), neo-debromoaplysiatoxin H (**2**) (4.3 mg).

### 2.1. Structure Elucidation of the New Compounds

**Figure 2:**
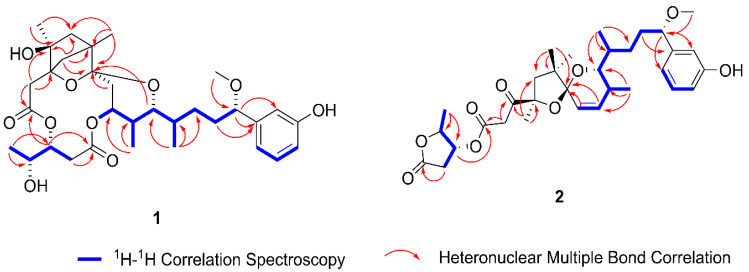
Key ^1^H-^1^H Correlation Spectroscopy and Heteronuclear Multiple Bond Correlations of **1** and **2**.

**Figure 3:**
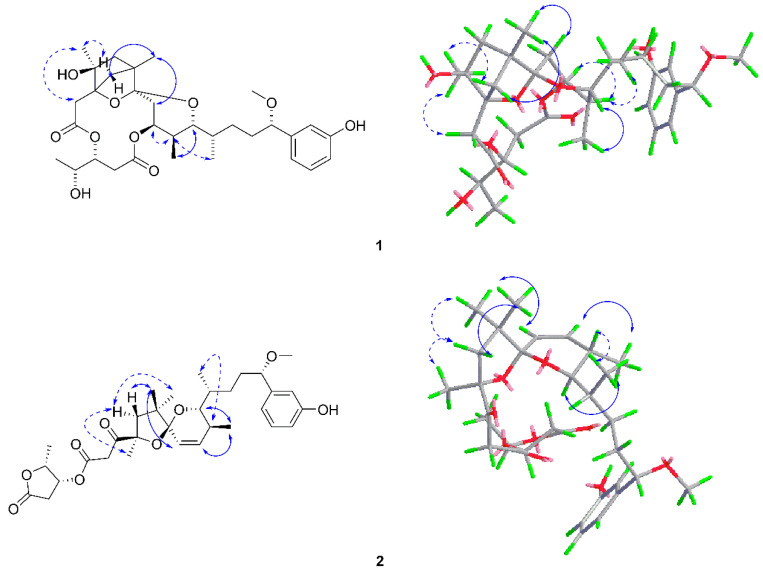
Key Nuclear Overhauser Effect Spectroscopy correlations of **1** and **2** (solid lines: α-orientation; dashed lines: β-orientation).

Neo-debromoaplysiatoxin G (**1**) was a white solid (${\left[\alpha \right]}_{D}^{25}$ + 31.83 (c 0.07, MeOH); UV (MeOH) λ_max_ (log ε) 217 (4.58), 275 (4.19) nm (Figure S2.9); IR (KBr) υ_max_ 3854, 3744, 3714, 2960, 1654, 1630, 1587 cm^−1^ (Figure S2.10); High-Resolution Electrospray Ionization Mass Spectroscopy (HRESIMS) data (*m*/*z* 613.2984 [M + Na]^+^, calcd 613.2989) (Figure S2.8) assign its molecular formula as C_32_H_46_O_10_ with ten degrees of unsaturation. The 32 carbon resonances can be accounted for as eight quaternary carbons, eleven methines, seven methylenes, and six methyls in ^13^C Nuclear Magnetic Resonance (NMR) (Table 1, Figures S2.1 and S2.2) and Distortionless Enhancement by Polarization Transfer (DEPT) spectra (Figure S2.3). Interpretation of the one-dimensional (1D) and two-dimensional (2D) NMR spectra (Figures S2.4–S2.7) indicated that the planar structure of **1** closely resembles those of aplysiatoxin analogues. Spectroscopic analysis indicated the presence of a side chain attached with a phenol ring at C-16, a methoxy at C-15, and a methyl at C-12 (C-12 to C-22), which was identical to the corresponding portion in ATXs. Heteronuclear Multiple Bond Correlation (HMBC) displayed there was a six-membered ring A connected C-1 (*δ* C 172.1) with methylene at C-2. Interestingly, HMBC correlations of H_2_-24 to C-4, C-5, C-6, C-7, and the slightly downfield chemical shifts of C-6 (*δ* C 48.7) explained the presence of the bridge ring (C3-C24-C6) on the ring A system. The ^13^C NMR chemical shifts of C-3 (*δ* C 85) and C-7 (δ C 105.5) showed having oxide functionalities on the six-membered bridged ring system. C-7 and C-11 were attached to oxide based on the 1D and 2D NMR shift data as well as the structural features of ATXs. Herein, the partially elucidated structure accounted for seven degrees of unsaturation. The ^1^H-^1^H Correlation Spectroscopy (COSY) correlations of H_2_-28/H-29/H-30/H_3_-31 and HMBC correlations of H-28 to C-27 (δ C 171.0), H-29 to C-27, H_3_-31 to C-29 (*δ* C 77.1), and C-30 (*δ* C 68.6) illustrated the existence of a portion of 3,4-dihydroxyvaleric acid (C-27−C-31), which was connected to C-2 methylene through an ester linkage with C-1 (*δ* C 172.1) and C-29 (*δ* C 77.1). According to the NMR data and the molecular formula of compound **1**, there remained one ring closure to complete the 10 degrees of unsaturation. A closer comparison of the NMR data of **1** and debromoaplysiatoxin suggested the presence of tetrahydropyran ring B. Although the ester linkage between C-9 and C-27 was not indicated by the HMBC spectrum, the ^1^H and ^13^C NMR data were strongly supportive of this remaining linkage, accounting for the final degree of unsaturation and thereby completing the closure of ring C. The planar structure of **1** was established with a novel structural skeleton featuring a bridge ring (C3-C24-C6) on the ring A system: bicyclo[2.1.1]tetrahydropyran as depicted in Figure 2. The Nuclear Overhauser Effect Spectroscopy (NOESY) experiment and vicinal coupling constants were utilized to speculate the relative configuration of **1** in Figure 3. The proton coupling constants 3.1 Hz of H-8a/H-9 and H-8b/H-9, as well as NOE correlations of H-9/H-10 and H-10/H_3_-23, which indicated H-9/H_3_-23 presenting an equatorial orientation on a 6-member ether ring. The H-10 and H-11 were determined as axial protontions by the large coupling constant *(J* = 10.7 Hz) of H-10/H-11. The NOE correlation of H-11/H-12 suggested a gauche conformer of H-11/H-12 based on previous aplysiatoxins [20]. The NOE correlation H-29/H-30 and coupling constants in accordance (*J* = 4.7 Hz) of H-29/H-28a, (*J* = 7.4 Hz) of H-29/H-28b with those of aplysiatoxins, demonstrated the stereochemistry of H-29 and H-30 was *syn* relationship [14]. The coupling constants (*J* = 8.2, 5.0 Hz) of H-15 and its chemical shift were similar with those of aplysiatoxins [20]. The absolute stereochemistry of C-3 and C-6 was determined by Gauge-Independent Atomic Orbital (GIAO) NMR shift calculation (Tables S1.4.1 and S1.4.2) followed by DP4^+^ analysis (Table S1.4.4) as isomer 2 (3*S*, 6*R*) with a probability of 100% based on ^1^H NMR, ^13^C NMR (Figure S1.4.1). Furthermore, due to their structural similarities, it was likely that compound **1** had a common biosynthetic origin with previously reported ATXs [14,20]. Therefore, the absolute stereochemistry was speculated as 3*S*, 4*S*, 6*R*, 9*S*, 10*S*, 11*R*, 12*S*, 15*S*, 29*R*, 30*R*.

Neo-debromoaplysiatoxin H (**2**), a colorless solid (${\left[\alpha \right]}_{D}^{25}$ + 31.55 (c 0.07, MeOH); UV (MeOH) λ_max_ (log ε) 217 (4.57), 274 (4.17) nm (Figure S2.19); IR (KBr) υ_max_ 3430, 1654, 1632, 1587, 1561, 1407, 1384 cm^−1^ (Figure S2.20), had a molecular formula of C_32_H_44_O_9_ with 11 degrees of unsaturation as established by the HRESIMS ion peak at *m/z* 595.2887 ([M + Na]^+^, calcd 595.2985) (Figure S2.18). Its ^13^C NMR and ^1^H NMR spectra (Figures S2.11 and S2.12) exhibited 32 carbon signals and attributed to one methoxy, six methyls, five methylenes, twelve methines, and eight quaternary carbons, including one carbonyl carbon (Table 1). The ^1^H NMR spectra and ^13^C NMR spectra revealed the molecule exhibited a 1, 3-disubstituted benzene ring, which was connected to the side chain at C-15 supported by the HMBC correlations (Figure S2.16) from H-15 to C-17 and C-21. Further, the γ-lactone ring was determined by consecutive ^1^H-^1^H COSY correlations (Figure S2.15) combined with the HMBC correlations, where C-27 and C-30 were possibly connected by an ester bond on basis of the ^1^H and ^13^C NMR shift data. The γ-lactone ring was connected to C-1 through an ester linkage with C-1and C-29 by the HMBC correlation from H-29 to C-1. The HMBC correlations from H_2_-2 to C-1, C-3 established the structure: β-ketone ester. HMBC cross-peaks, as well as the last unsaturation of this molecule explaining the unsaturated two dioxygen spiral ring (5/6) and the ring A (C4-C5-C6-C7), was connected to ring B (C8-C9-C10-C11) through C-7 (δ C 106.8). The C-4 was connected to C-7 and C-7 to C-11 through oxygen atom linkage with C-4 (δ C 86.2), C-7 (δ C 106.8), and C-11 (δ C 78.7). Accordingly, the planar structure of Compound **2** was confirmed (Figure 2), and the novel structural skeleton of neo-debromoaplysiatoxin H (**2**) was characterized with a unique 5/6 fused-dioxygen spiral ring system. The relative configuration of **2** was confirmed by the NOESY spectrum and vicinal coupling constants (Figure S2.17) (Figure 3). The small proton coupling constant (10.2 Hz) indicated an *Z* configuration of ∆^8,9^. The NOE correlation H_3_-26/H-5a/H_3_-24 and H-5b/H_3_-25 suggested that H-5b/H_3_-25 were α-orientation, H_3_-26/H-5a/H_3_-24 were β-orientation. Due to the NOE correlations of H_3_-25/H-8, H-9/H_3_-23/H-11, H-10/H_3_-22 and an *Z* configuration of ∆^8,9^, conferred that H_3_-23/H-11 were the same orientation, H_3_-22 in β-orientation. Because the signals of H-11/H-12 were overlapped with 15-OCH_3_ and H-14b, the coupling constants were not able to be obtained, and the relative stereochemistry of H-11/12 of **2** was proposed as 11R*, 12S*, with the same relative stereochemistry of **1** based on the same bio-genetics. The coupling constants of H and the chemical shifts of C and H at the γ-lactone ring in **2** were in accordance with those of 30-methylosciallatoxin D indicating the same relative configuration of **2** and the known ATXs at C-29 and C-30 [14,22], which established a relative stereochemistry of 29R* and 30R*. Therefore, the stereochemistry was speculated as 4R*, 7S*, 10S*, 11R*, 12S*, 15S*, 29R*, 30R*.

### 2.2. Biological Activities of the Isolated Compounds

#### 2.2.1. Inhibitory Activities against Kv1.5

**Figure 4:**
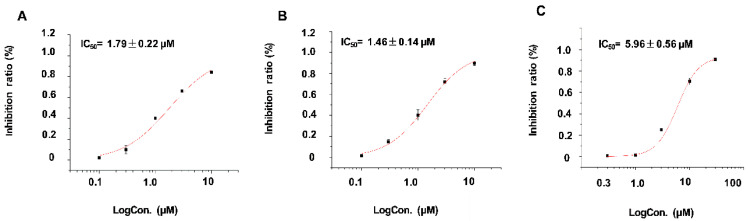
Dose-response study of **1** and **2** with Kv1.5 expression in Chinese hamster ovary (CHO) cells at holding potential (HP) of −80 mV. Data points represent mean ± SEM of 3 to 5 measurements. Solid curve fits to the Hill equation. (**A**) The inhibitory effect of 1 showed a half inhibitory concentration (IC_50_) value of 1.79 ± 0.22 μM; (**B**) The inhibitory effect of 2 showed an IC_50_ value of 1.46 ± 0.37 μM; (**C**) The inhibitory effect of acacetin showed an IC_50_ value of 5.96 ± 0.56 μM.

Ultra-rapid delayed rectifier K^+^ current (IKur) mediated by Kv1.5 is the main current in the repolarization process of cardiomyocyte action potentials, and potent blocking activity against potassium channel Kv1.5 may lead to the harmful effects on public health. In this study, we reported the ATXs isolated from Sanya, China, neo-debromoaplysiatoxin G (**1**) and neo-debromoaplysiatoxin H (**2**), whose inhibitory activities against Kv1.5 were evaluated (Figures S1.3.1.1–S1.3.1.3). Compounds **1** and **2** showed potent dose-response study results with half inhibitory concentration (IC_50_) values of 1.79 ± 0.22 µM and 1.46 ± 0.37 µM, respectively (Figure 4).

#### 2.2.2. Toxicity of Brine Shrimp

**Figure 5:**
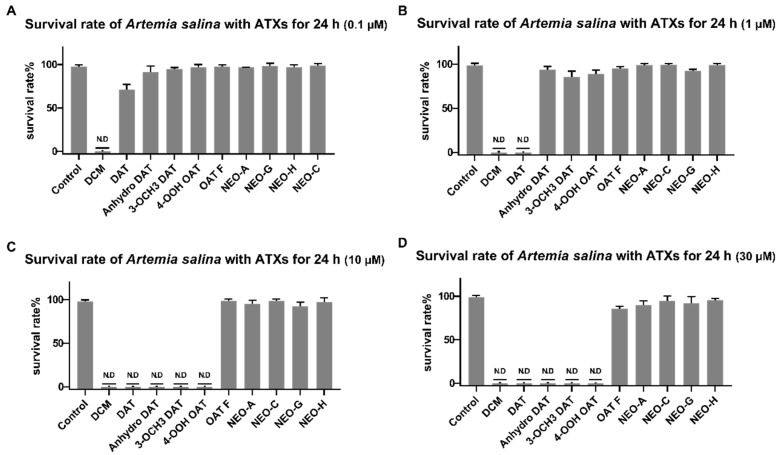
Effect of compound **1** and **2** to *Artemia salina* (*A. salina*). *A. salina* were treated with indicated concentration (0.1 µM, 1 µM, 10 µM, 30 µM) of dichloromethane (DCM), debromoaplysiatoxin (DAT), anhydrodebromoaplysiatoxin (Anhydro DAT), 3-methoxydebromoaplysaitoxin (3-OCH3 DAT), 4-hydroperoxyosciliatoxin B (4-OOH OAT), osciliatoxin F (OAT F), neo-debromoaplysiatoxin A (NEO-A), neo-debromoaplysiatoxin B (NEO-B), neo-debromoaplysiatoxin C (NEO-C), compound **1** and **2** for 24 h. The percentage of *A. salina* with all different kinds of ATXs. (**A**) The percentage of *A. salina* with all different kinds of aplysiatoxins (ATXs) in 0.1 µM; (**B**) the percentage of *A. salina* with all different kinds of ATXs in 1 µM; (**C**) the percentage of survival of *A. salina* with different kinds of ATXs in 10 µM; (**D**) the percentage of *A. salina* with all different kinds of ATXs in 30 µM. N.D: not detected the life of brine shrimp. The data were analyzed by GraphPad prism (Table S1.3.2.1).

The investigation of brine shrimp toxicity of nine aplysiatoxin derivatives (debromoaplysiatoxin (DAT), anhydrodebromoaplysiatoxin (Anhydro DAT), 3-methoxydebromoaplysaitoxin (3-OCH_3_ DAT), 4-hydroperoxyosciliatoxin B (4-OOH OAT), osciliatoxin F (OAT F), neo-debromoaplysiatoxin A (NEO-A), neo-debromoaplysiatoxin B (NEO-B), neo-debromoaplysiatoxin C (NEO-C), neo-debromoaplysiatoxin G (NEO-G), neo-debromoaplysiatoxin H (NEO-H) isolated from the same collection (Figure 1B), representing differential structural classifications was conducted. The survival of *Artemia salina* (*A. salina*) began to be influenced by DAT at the concentration as low as 0.1 µM as shown in Figure 5 (Table S1.3.2.1), followed by analogs of DAT (anhydro DAT, 3-OCH_3_ DAT) at a concentration of 10 µM. The other ATXs with different structural classifications such as OAT F, Neo-A, and compound **1** and **2** had no apparent effect at 30 µM. As the results indicated, debromoaplysiatoxin was the most toxic compound (IC_50_ value = 0.34 ± 0.036 µM) (Figure S1.3.2.2) compared to other tested derivatives, and the 3-hydroxy group at DAT seemed quite important to determine the higher toxicity in comparison to 3-OCH_3_ DAT and anhydro DAT, which are dehydroxylated or methylated of 3-hydroxy group at DAT, respectively.

## 3. Discussion

Species of *Lyngbya* as the major cyanobacterial species frequently detected from the occurrence of cyanobacterial blooms [28,29], can produce large array toxins, which cause irritant and allergenic responses in human and animal tissues with contact [2,30,31]. ATXs are a kind of dermatoxins from the marine cyanobacterium *Lyngbya* sp., which have drawn more and more attention due to their structural diversity associated with intriguing biological activities [11,15,16,17,18,32]. In this study, we reported the chemical and biological activities of two neo-ATXs along with many known ATXs isolated from this specimen. Based on their structural characteristics, the ATXs were classified into four categories: traditional ATXs with 6/12/6 tricyclic ring systems featuring a macrolactone ring (ABC ring); oscillatoxins featuring a spirobicyclic system (AB ring); nhatrangins with acyclic structure; and the fourth type, intriguing neo-ATXs displaying rare carbon skeletons differing that of traditional ATXs (Figure 1). Analysis of the distribution of 45 aplysiatoxin derivatives discovered across a relatively broad oceanic area (Figure S3.1, Table S3.1), showed that the traditional ATXs were most commonly detected compared to other ATXs, while the neo-ATXs were only discovered in the cyanobacterial samples from Hainan, China, and Okinawa, Japan. Whether multiple structural rearrangements of the traditional ATXs were caused by environmental stress, climate, water quality, or other factors remains to be further investigated.

In the previous reports, the traditional ATXs, such as ATX and DAT, exhibited multiple biological activities, correlated with skin irritation, diarrhea, antiviral activities, tumor-promoting, anti-proliferative, and pro-inflammatory [11,15,16,17,18]. Meanwhile, OAT I showed more potent cytotoxicity and diatom growth inhibition tests compared to the traditional ATXs such as ATX, DAT, 3-OCH_3_ DAT, and so on [14,23,33]. Most of neo-ATXs were found to have potent blocking action against potassium channel Kv1.5 (IC_50_ < 10 µM), in which NEO-B had the strongest inhibitory activity against Kv1.5 (IC_50_ = 0.30 ± 0.05 µM), and compound **1** and **2** had the similar inhibitory activity with NEO-E/F [20,21,33]. Interestingly, DAT and OAT E were also shown inhibitory activity against Kv1.5 (IC_50_ = 1.28 ± 0.08 µM, 0.79 ± 0.03 µM, respectively) [33]. In this study, the investigation of brine shrimp toxicity of nine aplysiatoxin derivatives showed that debromoaplysiatoxin was the most toxic compound. It should also be noted that the traditional ATXs displayed much higher activities in brine shrimp toxicity assay than other classified ATXs. Investigation of the correlation between the structure of the different ATX derivatives and their biological activities (cytotoxicity and brine shrimp toxicity) is ongoing.

Some ATXs such as DAT and NEO-A, structurally featuring a protein kinase C (PKC) recognition region (dilactone of ring C) and a conformational control region (6/6 Spiro ketal) [32], were reported to possess strong PKC activation as well as potassium channel Kv1.5 inhibition activities [20,21,34]. While most of neo-ATXs exhibited potent inhibition activities against potassium channel Kv1.5 without showing PKC activation [33], would this be the clue to explore the structure-activity relationship for the cause of lethal brine shrimp toxicity as well as the dermal toxicity of ATXs? Therefore, this work may provide extended information and knowledge for further understanding of ATXs with different structural modifications that may modulate multiple cellular signaling processes in animal systems leading to the harmful effects on public health.

In summary, two novel aplysiatoxin derivatives were isolated from the marine cyanobacterium *Lyngbya* sp. Neo-debromoaplysiatoxin G (**1**) and neo-debromoaplysiatoxin H (**2**) featuring novel structural skeletons characterized with a bicyclo[2.1.1]tetrahydropyran, and unique 5/6 fused-dioxygen spiral ring system, respectively. Compounds **1** and **2** showed potent blocking action against potassium channel Kv1.5 with IC_50_ values of 1.79 ± 0.22 µM and 1.46 ± 0.14 µM, respectively.

## 4. Materials and Methods

### 4.1. Material of Marine Cyanobacterium Lyngbya sp.

The cyanobacterium *Lyngbya* sp. was collected from the South China Sea in November 2016. The identification results of the samples were shown in Figure S1.1. A voucher specimen numbered as BNH-201606 had been well stored in Zhejiang Sci-Tech University.

### 4.2. General Experimental Procedures

The UV spectrum was obtained using Thermo UV/EV300 spectrophotometry. Optical rotations were recorded with a JASCO P-2000 polarimeter. ^1^H and ^13^C NMR spectra were measured on Agilent 600 MHz instruments using CDCL_3_ for chemical shifts. Chemical shifts (δ) were expressed in ppm regarding the solvent peak (H 7.26 and C 77.16). ESIMS and HRTOF-ESIMS data were recorded on Waters Xevo G2-XS QTOF spectrometer (Milford, MA, USA, Waters) after direct infusion. A Waters 1525 series instrument (Milford, MA, USA, Waters) equipped with Waters XBridge Prep C-18 column (5 μm, 10 mm × 250 mm, (Milford, MA, USA, Waters) and a 2998 photodiode array detector (Milford, MA, USA, Waters) was used for the high-performance liquid chromatography (HPLC) separation (Waters, Milford, MA, USA,). Silica gel (200–300) mesh, Yantai Jiangyou Silicone Development Co., Ltd. (Yantai, China) and Sephadex LH-20 (20–150 μm, Pharmacia, NJ, USA) were used for column chromatography. Methanol, chloroform, ethyl acetate, acetone, petroleum ether, and 2-propanol were purchased from Shanghai Chemical Reagents Co. (Shanghai, China). All other materials were of the highest grade available.

### 4.3. Extraction and Isolation

The freeze-dried powder of the Cyanobacterium (150 g) was extracted with CH_2_Cl_2_ /MeOH (1:1, *v*/*v*). The resultant extract was suspended in 1 L of MeOH/H_2_O (9:1, *v*/*v*) and partitioned with CH_2_Cl_2_ (3 × 1 L) to yield the CH_2_Cl_2_ extract (20 g), which was subjected to vacuum liquid chromatography (VLC) over silica gel using gradients of PE/EtOAc (5:1, 2:1, 1:1, 1:2, 1:5, 0:1, *v*/*v*) to obtain seven subfractions (F.A−G). F.D (800 mg) was further separated by reversed-phase octadecylsilyl silica (ODS) (10−100% MeCN/H_2_O, 180 min, flow rate 20 mL/min, UV detection at 190 nm) to afford twenty-one subfractions (F.D.1−21). Subsequently, the subfraction F.D.11 (55.4 mg) was purified by preparative HPLC (Waters SunFire Prep C18, 42% MeCN/H_2_O, 8.0 mL/min, UV detection at 190 nm) to yield neo-debromoaplysiatoxin G (**1**) (3.6 mg), subfraction F.D.17 (93.2 mg) was further separated by semi-preparative HPLC (YMC-Pack Pro C18, 85% MeOH/H_2_O, 3.0 mL/min, UV detection at 190 nm) to obtain neo-debromoaplysiatoxin H (**2**) (4.3 mg). Many reported ATXs also were obtained by the same collection (Supplementary Materials 1.2).

### 4.4. Ion Channel Inhibitory Experiment

Cell preparation: the day before the experiment, digestion of Chinese hamster ovary (CHO) cells (Sigma Chemical Co., St. Louis, MO, USA) with a density of 60–80% by trypsin, and split into some small glass plates, which placed in 35 mm petri dish, then added 10% fetal bovine serum (FBS) (Gibco, CA, USA), and Dulbecco’s modified eagle medium (DMEM) (HyClone, Logan, UT, USA) culture medium without P/S was cultured overnight in an incubator [35,36,37] (Supplementary Materials 1.3.1).

Electrophysiology: the cells were transferred to a perfusion tank and perfused with extracellular fluid. The intracellular fluid (mM) was: K Aspartate, 130; MgCl_2_, 5; EGTA 5; HEPES, 10; Tris-ATP 4; pH 7.2 (KOH titration). The intracellular fluid was stored in small portions in a refrigerator at −80 °C and thawed on the day of the experiment. The electrodes were filled with intracellular fluid and drawn with PC-10 (Narishige, Japan). Whole-cell patch-clamp recording, noise is filtered using one-fifth of the sampling frequency. The cells were clamped at −80 mV and then depolarized to 20 mV with a square wave lasting 2 s to obtain Kv1.5 current. This procedure is repeated every 20 s. After it was stabilized, compound **1**, compound **2**, and acacetin were perfused, and when the reaction was stabilized, the strength of the blocking was calculated (Supplementary Materials 1.3.1).

### 4.5. Brine Shrimp Toxicity Assay

The lethality assay of brine shrimp *A. salina* was developed by Vanhaecke et al. [38]. This assay had also been suggested to evaluate the toxicity of compounds [39,40,41]. Commercially available *A. salina* or brine shrimp cysts were purchased and cultivated in 3.2% of saline water. Before cultivation, the saline was aerated, and then cysts were kept at room temperature for 24 h. For toxicity screening, hatched larvae were collected and introduced in saline water. Add 0.9% saline water and approximately the same number of larvae 30 per well to make a 96-wells test culture plate. ATXs with 0.1 μM, 1 μM, 10 μM, 30 μM were added to the test culture plate, while the equal volume of dimethyl sulfoxide (DMSO) (Aladdin, Shanghai, China) and dichloromethane (Aladdin, Shanghai, China) were added as blank control test and positive control separately. After 24 h at 25 °C, the percent of survival of *A. salina* was calculated (Supplementary Materials 1.3.2).

## Figures and Tables

**Table 1:** ^1^H (600 MHz) and ^13^C (150 MHz) NMR Data for Compounds **1**, **2** in CDCL_3_ (δ in ppm, *J* in Hz).

Pos.	1		2	
	δ_H_ (*J* in Hz)	δ_C_	δ_H_ (*J* in Hz)	δ_C_
1		172.1		167.4
2	a 2.72, d (12.6) b 2.69, d (12.6)	34.8	a 4.10, d (16.5) b 3.45, d (16.5)	43.5
3		85		206.5
4		79.2		86.2
5	a 2.02, d (10.5) b 1.84, d (10.5, 2.9)	47.1	a 2.59, d (12.7) b 1.68, d (12.7)	46.4
6		48.7		47,3
7		105.5		106.8
8	a 2.24, dd (14.3, 3.1) b 1.59, dd (14.3, 3.2)	34	5.60, dd (10.2, 2.7)	122.6
9	4.89, m	74.1	5.86, dd (10.2, 2.7)	138.1
10	1.69, m	34	2.16, m	30.0
11	3.86, d (10.7)	74	3.21, overlap	78.7
12	1.52, m	33.9	1.60, overlap	34.6
13	a 1.52, m b 1.41, m	31.3	1.27	29.4
14	a 1.79, m b 1.62, m	37.5	a 1.85, m b 1.60, overlap	36.3
15	4.00, dd (8.2, 5.0)	84.6	3.94, t-like (6.7)	83.5
16		144.7		144
17	6.79, t-like (2.0)	113.5	6.77, overlap	113.9
18		156.3		156.1
19	6.76, ddd (8.0, 2.6, 1.2)	114.7	6.77, overlap	114.6
20	7.22, t-like (8.0)	129.8	7.21, t-like (7.7)	129.7
21	6.86, dt (8.0, 1.2)	118.2	6.83, d (7.4)	118.5
22	0.81, d (6.4)	12.1	0.81, d (6.8)	13.3
23	0.78, d (6.9)	13.6	0.90, d (6.4)	17.0
24	a 2.47, dd (12.9, 2.9) b 1.31, d (12.9)	47.2	0.89, s	23.0
25	1.01, s	15.7	1.09, s	26.3
26	1.41, s	22.3	1.43, s	25.7
27		171		174.3
28	a 2.95, dd (14.8, 4.7) b 2.56, d (14.8, 7.4)	36.3	a 2.92, dd (18.3, 6.1) b 2.70, d (18.3)	36.7
29	4.81, m	77.1	5.51, t-like (5.1)	72.1
30	4.22, m	68.6	4.73, m	79.1
31	1.23, d (6.4)	18.5	1.39, d (6.6)	14.1
15-OCH_3_	3.26, s	57.2	3.21, overlap	56.6

NMR data of debromoaplysiatoxin in Table S3.2 of Supplementary Materials.

## Supplementary Materials

The following are available online at https://www.mdpi.com/2072-6651/12/11/733/s1, experimental details, Figure S1.1: identification of cyanobacterium; Figure S1.3.1: ion channel experiment; Figure S1.3.2: brine shrimp cytotoxicity assay; Figures S2.1–S2.20: 1D and 2D NMR, HRESIMS, UV and IR spectra of compounds **1** and **2**; Figure S1.4.1 and Tables S1.4.2–S1.4.4: NMR calculation and followed by DP4^+^ analysis of **1**; Figure S3.1 and Table S3.1: the distribution of four types of ATXs in various locations.
