# Supplementary material for: Chemical and Biological Study of Novel Aplysiatoxin Derivatives from the Marine Cyanobacterium Lyngbya sp
Source: Toxins (Basel). 2020 Nov 23;12(11):733. doi: 10.3390/toxins12110733 (PMC7700248; doi:10.3390/toxins12110733)
Supplement: Supplementary file 1 [file toxins-12-00733-s001.pdf]

# Supplementary Materials: Chemical and Biological Study of Novel Aplysiatoxin Derivatives from the Marine Cyanobacterium *Lyngbya* sp.

**Hui-Hui Zhang, Xin-Kai Zhang, Ran-Ran Si, Si-Cheng Shen, Ting-Ting Liang, Ting-Ting Fan, Wei Chen, Lian-Hua Xu and Bing-Nan Han**

## 1. Experimental Details

### *1.1. Morphological and Molecular Identification of Cyanobacterium*

The cyanobacterium strain used in this study were collected from Harbor of Hainan Sanya, China, Named as cyanobacterium HN. Colonies of cyanobacterium HN appeared as dark red, brown, or black tufts ranging from 15 to 25 cm in length and grew attached to sea rock and surface of the sea.

Filament width, cell width, and cell length of cyanobacterium HN were measured on the compound light microscope (Zeiss, Germany) with a 20× objective and 10× ocular lens with a calibrated optical micrometer. Filaments were long, of indeterminate length, 55–65 μm wide, formed by a uniseriate row of discoid cells encased in a firm, colorless, hyaline sheath which, when old, became yellowed and distinctly lamellated. Cells were discoid, 6–8 μm long, 30–40 μm broad, with rounded end cells without calyptra. Cell contents were finely granular without prominent granular inclusions (Figure S1.1A).

16S rDNA was used to characterize the identity of cyanobacterium samples. Total cyanobacterium genomic DNA from lyophilized samples was extracted by using TianGen Plant Genomic DNA Kit (TIANGEN Biotech Co., Ltd., Beijing, China) according to the manufacturer's instructions. Three PCR primer sets, CYA106F (5′-TACGGCTACCTTGTTAACGCGTGA-3′)/781R (5′-GACTACTGGGGTATC-TAATCCCATT-3′), 27F (5′-AGAGTTTGATCCTGGCTCAG-3′)/809R (5′-GC-TTCGGCACGGCTCGGGTCGATA-3′) and MSR2F (5′-CGGTAATACGGGG-GAGGCAA-3′)/2R (5′-CCAACATCTCACGACACGAG-3′), were used for amplifying 16S rDNA. PCR reactions were performed in a BIO-RAD Cycler C1000, according to the following profile: 5 min at 95 °C and 35 cycles of 30 s at 95°C, 1 min at 58 °C for CYA106F/781R, 30 s at 50 °C for 27F/809R or MSR2F/2R, and 1 min at 72 °C, followed by 10 min at 72 °C. The products were analyzed by electrophoresis in 0.7% (*w*/*v*) agarose gels electrophoresis. 16S rDNA sequences of other cyanobacterial taxa were acquired from NCBI GenBank and EzBioCloud databases and aligned by using MUSCLE implemented in MEGA7.0, and the phylogenetic tree was reconstructed by MrBayes.

Cyanobacterium HN held the highest 16S rRNA gene similarity with *Lyngbya* sp. CENA128$^T$ with the value of 99%, revealing that cyanobacterium HN might belong to *Lyngbya* sp. The phylogenetic trees based on the 16S rRNA gene sequences, reconstructed with the Bayesian MCMC methods, showed that cyanobacterium HN fell into the clade comprising *Lyngbya* species and formed a stable clade with *Lyngbya* sp. CENA128$^T$ (Figure S1.1B). According to these results, cyanobacterium HN belonged to *Lyngbya* sp.

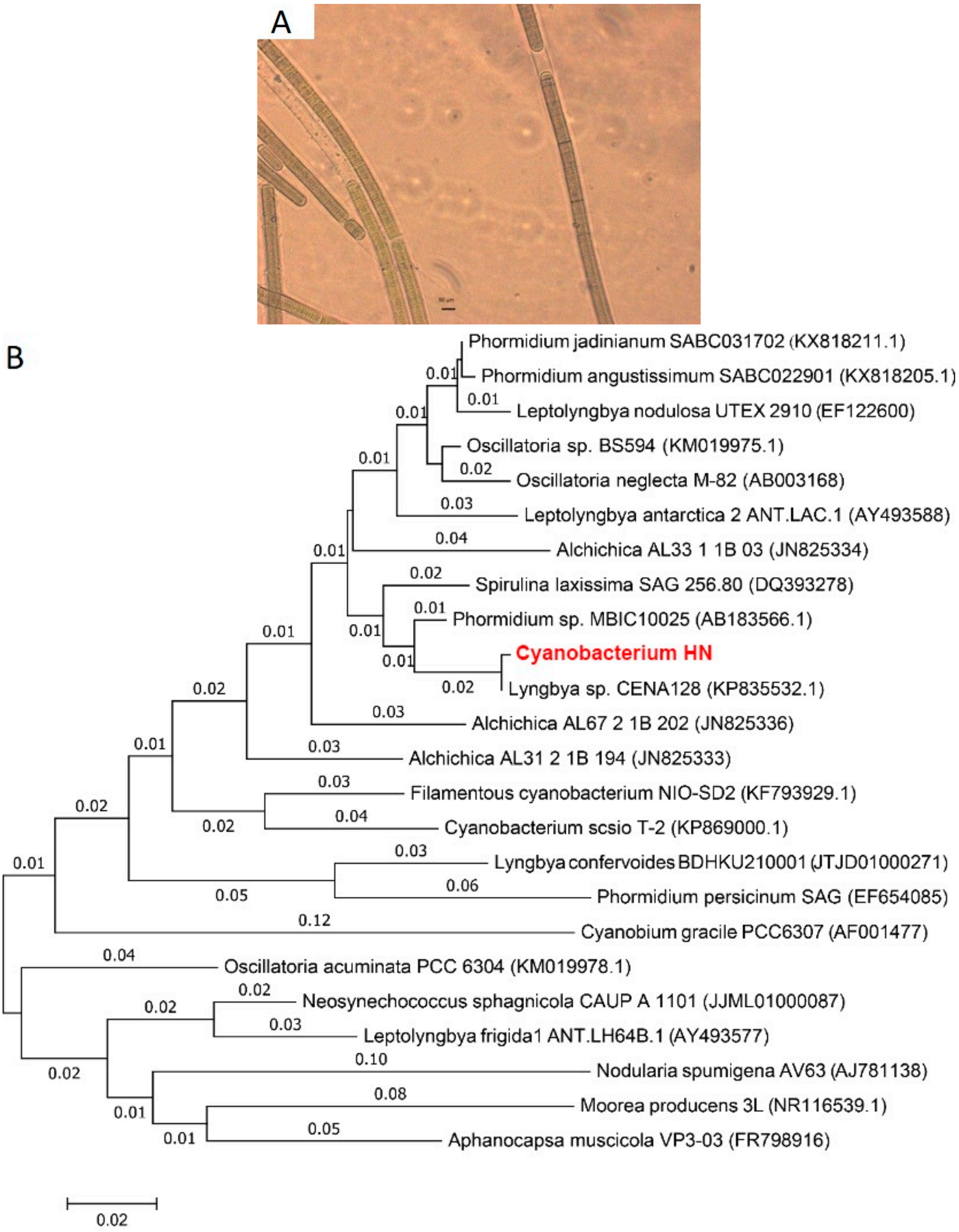


**Figure S1.1.** (**A**) *Lyngbya* sp. collected in Harbor of Hainan. The specie, as identified based on morphological features, is shown in light micrographs. (**B**) Bayesian phylogenetic tree of *Lyngbya* sp. HN and its close relatives. Bootstrap was set as 20,000 replicates. Bar, 0.02 substitutions per nucleotide position.

## *1.2. Extraction and Isolation*

The freeze-dried powder of the Cyanobacterium (150 g) was extracted with $CH_2Cl_2$ /MeOH (1:1, *v*/*v*). The resultant extract was suspended in 1 L of MeOH/$H_2O$ (9:1, *v*/*v*) and partitioned with $CH_2Cl_2$ (3 × 1 L) to yield the $CH_2Cl_2$ extract (20 g), which was subjected to VLC over silica gel using gradients of PE/EtOAc (5:1, 2:1, 1:1, 1:2, 1:5, 0:1, *v*/*v*) to obtain seven subfractions (F.A-G). F.D (800 mg) was further separated by reversed-phase ODS (10–100% MeCN/$H_2O$, 180 min, flow rate 20 mL/min, UV

detection at 190 nm) to afford twenty-one subfractions (F.D.1-21). Subsequently, the subfraction F.D.11 (55.4 mg) was purified by preparative HPLC (Waters SunFire Prep C18, 42% MeCN/$H_2O$, 8.0 mL/min, UV detection at 190 nm) to yield neo-debromoaplysiatoxin G (**1**) (3.6 mg), subfraction F.D.17 (93.2 mg) was further separated by semi-preparative HPLC (YMC-Pack Pro C18, 85% MeOH/$H_2O$, 3.0 mL/min, UV detection at 190 nm) to obtain neo-debromoaplysiatoxin H (**2**) (4.3 mg). Neo-debromoaplysiatoxin A, neo-debromoaplysiatoxin B, neo-debromoaplysiatoxin C, neo-debromoaplysiatoxin D, neo-debromoaplysiatoxin E, neo-debromoaplysiatoxin F, debromoaplysiatoxin, 3-methoxydebromoaplysiatoxin, anhydrodebromoaplysiatoxin, 4-hydroperoxyoscillatoxin B, oscillatoxin E and oscillatoxin F also were obtained from the same collection.

### *1.3. Bioassays*

#### 1.3.1. Ion Channel Experiment

**Cell preparation.** One day before the experiment, the density of 60–80% CHO cells (Sigma Chemical Co., St. Louis, MO, USA) was digested with trypsin and placed in DMEM medium without P/S, added After 10% FBS, it was cultured overnight in an incubator.

**Electrophysiology.** The cells were transferred to a perfusion tank and perfused with extracellular fluid. The intracellular fluid (mM) was: K Aspartate, 130; $MgCl_2$, 5; EGTA 5; HEPES, 10; Tris-ATP 4; pH 7.2 (KOH titration). The intracellular fluid was stored in small portions in a refrigerator at −80 °C and thawed on the day of the experiment. The electrodes were filled with intracellular fluid and drawn with PC-10 (Narishige, Japan). Whole-cell patch-clamp recording, noise is filtered using one-fifth of the sampling frequency. The cells were clamped at –80 mV and then depolarized to 20 mV with a square wave lasting 2 s to obtain Kv1.5 current. This procedure is repeated every 20 s. After it was stabilized, compound **1**, compound **2,** and acacetin were perfused, and when the reaction was stabilized, the strength of the blocking was calculated.

**Data analysis and statistics.** Data acquisition and analysis were carried out using pCLAMP 10 (Molecular Devices, Union City, CA). Data fitting and statistical analyses were performed using ORIGIN 8.0 (GraphPad Software Inc., San Diego, CA). The $IC_{50}$ value was determined by fitting the data points to the equation. Where $IC_{50}$ is the concentration at which half-maximal currents were inhibited, all the data were presented as mean ± SEM.

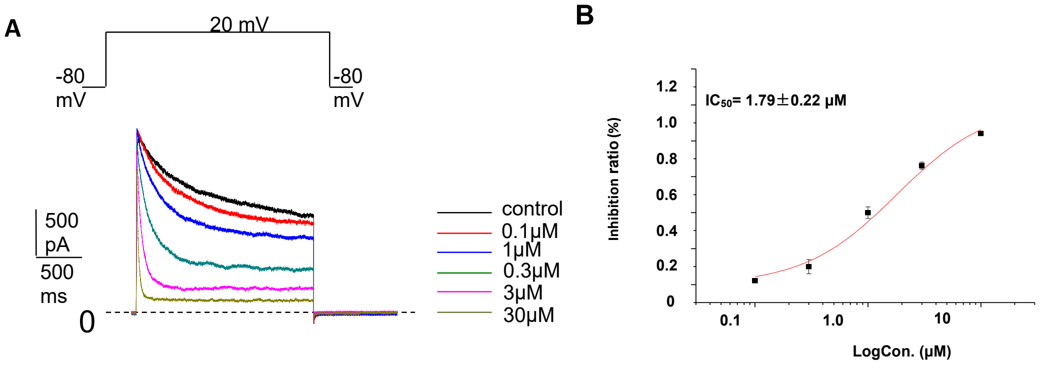


**Figure S1.3.1.1.** (**A**) Kv1.5 traces elicited by 5 s pulses from −80 to + 20 mV and tail currents recorded at −80 mV in the absence and presence of 0.1 μM, 0.3 μM, 1 μM, 3 μM, 10 μM, and 30 μM neo-debromoaplysiatoxin G (**1**). (**B**) Percent blocked-Concentration curves. The abscissa represents the concentration, and the ordinate represents the percentage of Kv1.5 current that is blocked at different concentrations of neo-debromoaplysiatoxin G (**1**). Data points represent mean ± SEM of 3 to 5 measurements, and the inhibitory effect showed an $IC_{50}$ value of 1.79 ± 0.22 μM.

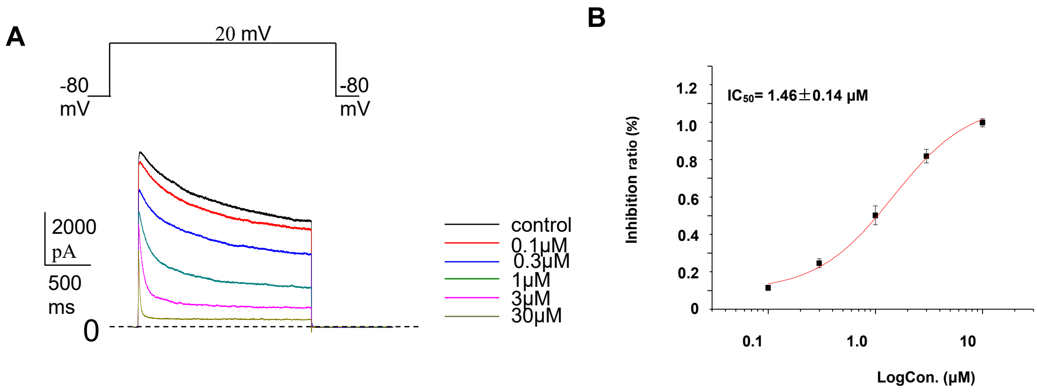


**Figure S1.3.1.2.** (**A**) Kv1.5 traces elicited by 5 s pulses from −80 to + 20 mV and tail currents recorded at −80 mV in the absence and presence of 0.1 μM, 0.3 μM, 1 μM, 3 μM, 10 μM and 30 μM neo-debromoaplysiatoxin H (**2**). (**B**) Percent blocked-concentration curves. The abscissa represents the concentration, and the ordinate represents the percentage of Kv1.5 current that is blocked at different concentrations of neo-debromoaplysiatoxin H (**2**). Data points represent the mean ± SEM of 3 to 5 measurements, and the inhibitory effect showed an $IC_{50}$ value of 1.46 ± 0.14 μM.

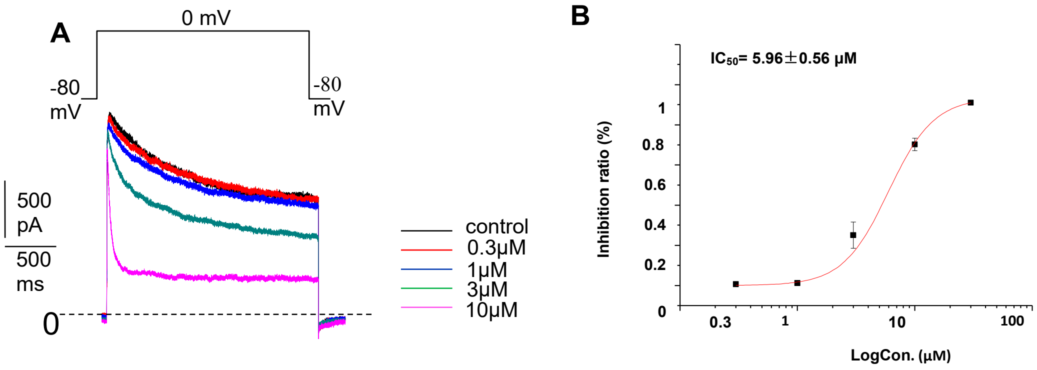


**Figure S1.3.1.3.** (**A**) Kv1.5 traces elicited by 5 s pulses from −80 to + 20 mV and tail currents recorded at −80 mV in the absence and presence of 0.3 μM, 1 μM, 3 μM, 10 μM, and 30 μM acacetin. (**B**) Percent blocked-concentration curves. The abscissa represents the concentration, and the ordinate represents the percentage of Kv1.5 current that is blocked at different concentrations of acacetin. Data points represent mean ± SEM of 3 to 5 measurements, and the inhibitory effect showed an $IC_{50}$ value of 5.96 ± 0.56 μM.

### 1.3.2. Brine Shrimp Cytotoxicity Assay

Commercially available *Artemia salina* (*A. salina)* or brine shrimp cysts were purchased and cultivated in 3.2% of saline water. Before cultivation, the saline was aerated, and then cysts were kept at room temperature for 24 h. For toxicity screening, hatched larvae were collected and introduced in saline water. Add saline water and equivalent larvae in per well of 96 wells to make test culture plate. Aplysiatoxins with 0.1 μM, 1 μM, 10 μM, 30 μM was added to test culture plate, while DMSO and dichloromethane were added as blank control test and positive control separately. After 24 h in 25 °C, the percent of survival of *A. salina* was calculated.

**Table S1.3.2.1**. Effect of compound **1** and **2** to *Artemia salina (A. salina)*. *A. salina* were treated with indicated concentration (0.1 μM, 1 μM, 10 μM, 30 μM) of dichloromethane (DCM), debromoaplysiatoxin (DAT), anhydrodebromoaplysiatoxin (Anhydro DAT), 3-methoxydebromoaplysaitoxin (3-$OCH_3$ DAT), 4-hydroperoxyosciliatoxin B (4-OOH OAT), osciliatoxin F (OAT F), neo-debromoaplysiatoxin A (NEO-A), neo-debromoaplysiatoxin B (NEO-B), neo-debromoaplysiatoxin C (NEO-C), compound **1** and **2** for 24 h. The percentage of *A. salina* with all different kinds of aplysiatoxins (ATXs).

| **Compounds** | **Percentage of Survival of *Artemia salina* after 24 h（μM）** | | | |
|---|---|---|---|---|
| | **0.1** | **1** | **10** | **30** |
| Debromoaplysiatoxin | 70.88 ± 6.30 | 0 | 0 | 0 |
| Anhydrodebromoaplysiatoxin | 93.98 ± 3.46 | 91.34 ± 6.94 | 0 | 0 |
| 3-methoxydebromoaplysiatoxin | 94.46 ± 1.87 | 85.82 ± 6.30 | 0 | 0 |
| 4-hydroperoxyoscillatoxin B2 | 96.77 ± 3.23 | 88.92 ± 4.38 | 0 | 0 |
| Oscillatoxin F | 97.50 ± 2.20 | 98.85 ± 1.99 | 95.32 ± 1.92 | 85.54 ± 2.91 |
| Neo-debromoaplysiatoxin A | 96.50 ± 0.59 | 98.89 ± 1.92 | 95.25 ± 4.21 | 89.97 ± 4.74 |
| Neo-debromoaplysiatoxin C | 98.67 ± 2.31 | 99.05 ± 1.65 | 98.89 ± 1.92 | 94.90 ± 5.27 |
| Neo-debromoaplysiatoxin G | 98.04 ± 3.40 | 92.57 ± 1.54 | 92.39 ± 4.71 | 91.84 ± 7.73 |
| Neo-debromoaplysiatoxin H | 97.27 ± 2.38 | 98.88 ± 1.93 | 97.33 ± 4.62 | 95.53 ± 1.93 |
| Methylenechloride | | 0 | 0 | 0 |
| Control | 97.78 ± 1.93 | 98.77 ± 2.13 | 97.74 ± 1.96 | 98.77 ± 2.14 |

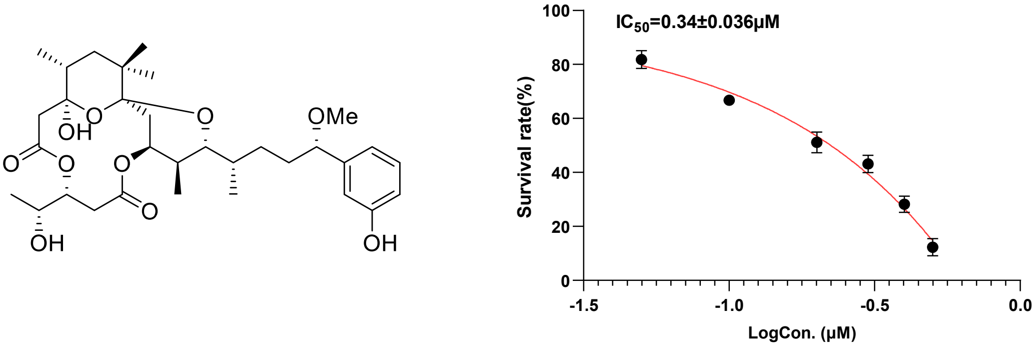


**Figure S1.3.2.1.** Effect of debromoaplysiatoxin to *Artemia salina (A. salina)*. *A. salina* were treated with debromoaplysiatoxin with indicated concentration 0.05 μM, 0.1 μM, 0.2 μM, 0.3 μM, 0.4 μM, 0.5 μM and inhibitory effect showed $IC_{50}$ value of 0.034 ± 0.036 μM.

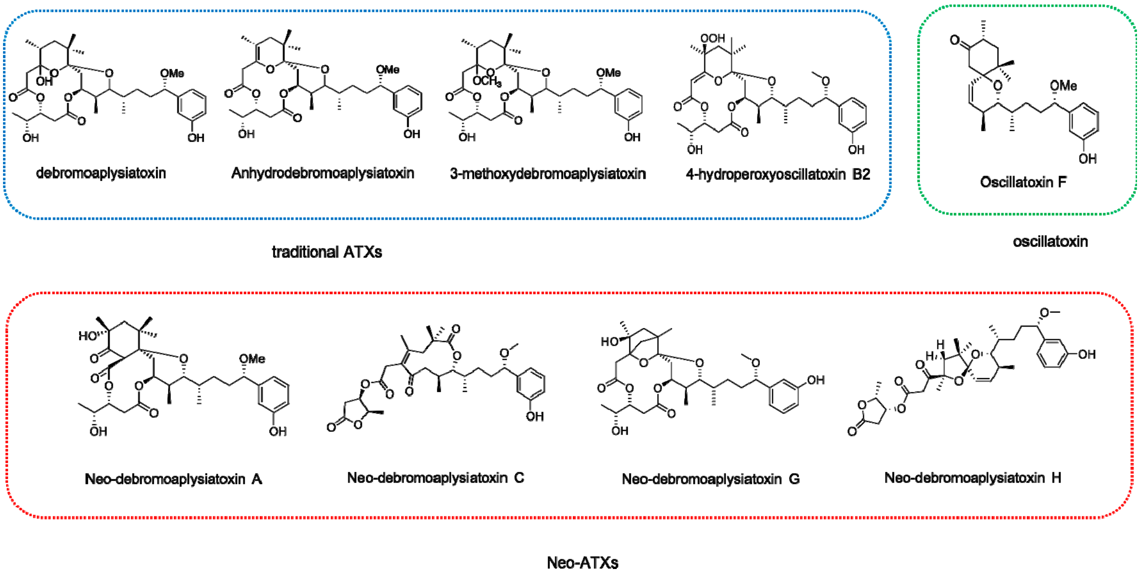

**Figure S1.3.2.2**. Structures of different types of aplysiatoxins to brine shrimp toxicity assay.

## *1.4. Methods for NMR Calculation of Neo-Debromoaplysiatoxin G (1)*

Monte Carlo conformational searches were carried out by means of the Spartan's 10 software (Spartan Software, San Francisco, CA, USA) using Merck Molecular Force Field (MMFF). The conformers with Boltzmann-population of over 1% were chosen for NMR calculations, and then the conformers were initially optimized at B3LYP/6–31g (d, p) level in gas. Meanwhile, gauge-independent atomic orbital (GIAO) calculations of $^{1}H$ and $^{13}C$ NMR chemical shifts were accomplished by density functional theory (DFT) at the mPWLPW91-SCRF (methanol)/6–311+g (d, p) level with the PCM solvent continuum model in Gaussian 09 software (Gaussian, Wallingford, CT, USA). The calculated NMR data of the lowest energy conformers for isomer 1, isomer 2 were averaged according to the Boltzmann distribution theory and their relative Gibbs free energy. The $^{1}H$ and $^{13}C$ NMR chemical shifts for TMS were calculated by the same protocol and used as reference. The experimental and calculated data were analyzed by the improved probability DP4$^{+}$ method for isomeric compounds. A significant higher DP4$^{+}$ probability score suggested the correctness of its configuration.

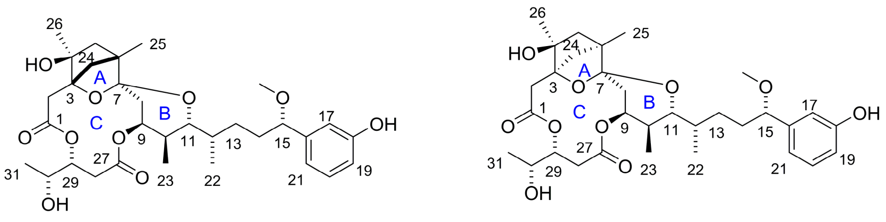


Isomer 1 Isomer 2

**Figure S1.4.1.** isomer 1 (3R, 6S) and isomer 2 (3S, 6R ) of neo-debromoaplysiatoxin G (**1**).

**Table S1.4.1.a.** Gibbs free energies$^{a}$ and equilibrium populations$^{b}$ of low-energy conformers of isomer 1.

| Conformers | In MeOH | |
|---|---|---|
| | Δ*G* | ***P*** **(%)** |
| isomer 1-1 | 0 | 62.82 |
| isomer 1-2 | 1.77 | 5.23 |
| isomer 1-3 | 0.38 | 23.18 |
| isomer 1-4 | 1.65 | 8.78 |

$^{a}$B3LYP/6–31+G(d, p), in kcal/mol. $^{b}$From Δ*G* values at 298.15 K.

**Table S1.4.1.b.** Cartesian coordinates for the low-energy reoptimized MMFF conformers of isomer 1 at B3LYP/6–31G (d, p) level of theory in $CH_3OH$.

| Isomer 1-1 | | | Standard Orientation (Ångstroms) | | |
|---|---|---|---|---|---|
| **Center Number** | **Atomic Number** | **Atomic Type** | **X** | Y | Z |
| 1. | 6. | 0. | 4.047422 | 1.995133 | 0.487903 |
| 2. | 6. | 0. | 4.880462 | 1.592217 | 1.542568 |
| 3. | 6. | 0. | 5.389948 | 0.297816 | 1.559078 |
| 4. | 6. | 0. | 5.082094 | −0.611455 | 0.542304 |
| 5. | 6. | 0. | 4.255568 | −0.214943 | −0.513626 |
| 6. | 6. | 0. | 3.746161 | 1.088362 | −0.537320 |
| 7. | 6. | 0. | 3.859563 | −1.182160 | −1.626720 |
| 8. | 8. | 0. | 3.574934 | 3.267349 | 0.501653 |
| 9. | 6. | 0. | 2.455209 | −1.778798 | −1.423483 |
| 10. | 8. | 0. | 4.766216 | −2.271954 | −1.761597 |

| | | | | | |
|---|---|---|---|---|---|
| 11. | 6. | 0. | 5.983568 | −1.928749 | −2.398860 |
| 12. | 6. | 0. | 2.317400 | −2.687523 | −0.194300 |
| 13. | 6. | 0. | 0.869960 | −3.172364 | 0.045533 |
| 14. | 6. | 0. | 0.819608 | −4.480665 | 0.849031 |
| 15. | 6. | 0. | −0.067015 | −2.074693 | 0.607739 |
| 16. | 6. | 0. | −0.129531 | −1.906447 | 2.145356 |
| 17. | 6. | 0. | −1.248178 | −0.913240 | 2.498987 |
| 18. | 6. | 0. | 1.197813 | −1.502528 | 2.802171 |
| 19. | 6. | 0. | −2.436714 | −1.526214 | 0.357586 |
| 20. | 8. | 0. | −0.782001 | 0.394873 | 2.034075 |
| 21. | 6. | 0. | −2.268825 | 0.944694 | −2.330839 |
| 22. | 6. | 0. | −2.969979 | −0.069024 | −1.406223 |
| 23. | 6. | 0. | −4.414123 | 0.301919 | −0.941510 |
| 24. | 6. | 0. | −4.837818 | −1.033089 | −0.242182 |
| 25. | 6. | 0. | −3.652828 | −2.009961 | −0.488070 |
| 26. | 6. | 0. | −1.061199 | 1.594448 | −1.685532 |
| 27. | 8. | 0. | 0.074590 | 1.486587 | −2.125076 |
| 28. | 8. | 0. | −1.378936 | −2.429075 | 0.104219 |
| 29. | 6. | 0. | −3.999726 | −3.484173 | −0.341877 |
| 30. | 8. | 0. | −5.142026 | 0.506070 | −2.163854 |
| 31. | 6. | 0. | −2.583358 | −1.331110 | 1.865857 |
| 32. | 8. | 0. | −1.364604 | 2.323319 | −0.597638 |
| 33. | 6. | 0. | −1.518989 | 1.482029 | 2.303171 |
| 34. | 8. | 0. | −2.525283 | 1.481544 | 2.983333 |
| 35. | 6. | 0. | −0.917909 | 2.756437 | 1.719958 |
| 36. | 6. | 0. | −0.282719 | 2.651812 | 0.333029 |
| 37. | 6. | 0. | 0.456812 | 3.939775 | −0.082115 |
| 38. | 6. | 0. | −0.465907 | 5.086112 | −0.492071 |
| 39. | 8. | 0. | 1.414986 | 3.677537 | −1.116658 |
| 40. | 6. | 0. | −3.163702 | −1.517072 | −1.877373 |
| 41. | 8. | 0. | −2.167387 | −0.233851 | −0.215883 |
| 42. | 6. | 0. | −4.541502 | 1.538327 | −0.058696 |
| 43. | 1. | 0. | 5.114838 | 2.306528 | 2.325076 |
| 44. | 1. | 0. | 6.037278 | −0.009134 | 2.376360 |
| 45. | 1. | 0. | 5.478596 | −1.620542 | 0.557563 |
| 46. | 1. | 0. | 3.109731 | 1.405050 | −1.359814 |
| 47. | 1. | 0. | 3.838506 | −0.615822 | −2.573465 |
| 48. | 1. | 0. | 2.942086 | 3.401970 | −0.239026 |
| 49. | 1. | 0. | 1.746981 | −0.943333 | −1.377478 |
| 50. | 1. | 0. | 2.205582 | −2.352843 | −2.324773 |
| 51. | 1. | 0. | 6.549173 | −1.175457 | −1.834481 |
| 52. | 1. | 0. | 6.578097 | −2.843277 | −2.465929 |
| 53. | 1. | 0. | 5.808918 | −1.543540 | −3.415404 |
| 54. | 1. | 0. | 2.706210 | −2.174309 | 0.689678 |
| 55. | 1. | 0. | 2.960353 | −3.561747 | −0.342914 |
| 56. | 1. | 0. | 0.441760 | −3.393002 | −0.941368 |
| 57. | 1. | 0. | 1.308966 | −4.389397 | 1.825067 |
| 58. | 1. | 0. | −0.213729 | −4.801552 | 1.014125 |
| 59. | 1. | 0. | 1.333935 | −5.278095 | 0.303396 |
| 60. | 1. | 0. | 0.210291 | −1.109231 | 0.168993 |
| 61. | 1. | 0. | −0.450859 | −2.866472 | 2.569423 |
| 62. | 1. | 0. | −1.370416 | −0.840782 | 3.584853 |
| 63. | 1. | 0. | 1.935437 | −2.305686 | 2.748495 |
| 64. | 1. | 0. | 1.629949 | −0.614212 | 2.334335 |
| 65. | 1. | 0. | 1.038200 | −1.275181 | 3.862274 |
| 66. | 1. | 0. | −1.909481 | 0.459456 | −3.239406 |
| 67. | 1. | 0. | −2.983439 | 1.718474 | −2.628345 |
| 68. | 1. | 0. | −5.733838 | −1.422637 | −0.735266 |

| | | | | | |
|---|---|---|---|---|---|
| 69. | 1. | 0. | −5.074460 | −0.904545 | 0.817618 |
| 70. | 1. | 0. | −3.126100 | −4.111394 | −0.530548 |
| 71. | 1. | 0. | −4.788634 | −3.762512 | −1.048367 |
| 72. | 1. | 0. | −4.367504 | −3.708104 | 0.666267 |
| 73. | 1. | 0. | −6.024954 | 0.822614 | −1.930928 |
| 74. | 1. | 0. | −2.866852 | −2.287338 | 2.320248 |
| 75. | 1. | 0. | −3.351952 | −0.600376 | 2.109885 |
| 76. | 1. | 0. | −1.707527 | 3.510351 | 1.738932 |
| 77. | 1. | 0. | −0.138887 | 3.083392 | 2.420145 |
| 78. | 1. | 0. | 0.430802 | 1.824923 | 0.316069 |
| 79. | 1. | 0. | 1.051430 | 4.246458 | 0.786833 |
| 80. | 1. | 0. | −1.134309 | 5.376311 | 0.325238 |
| 81. | 1. | 0. | −1.078821 | 4.806128 | −1.352981 |
| 82. | 1. | 0. | 0.138838 | 5.953528 | −0.766271 |
| 83. | 1. | 0. | 1.078215 | 2.933627 | −1.657028 |
| 84. | 1. | 0. | −2.230783 | −1.986863 | −2.192554 |
| 85. | 1. | 0. | −3.924654 | −1.606578 | −2.654885 |
| 86. | 1. | 0. | −4.199026 | 2.433211 | −0.585628 |
| 87. | 1. | 0. | −3.975750 | 1.446475 | 0.868363 |
| 88. | 1. | 0. | −5.595524 | 1.687028 | 0.210204 |

| **Isomer 1-2** | | | **Standard Orientation (Ångstroms)** | | |
|---|---|---|---|---|---|
| **Center Number** | **Atomic Number** | **Atomic Type** | **X** | Y | Z |
| 1. | 6. | 0. | 4.071921 | 2.107031 | 0.410127 |
| 2. | 6. | 0. | 5.027380 | 1.824747 | 1.397574 |
| 3. | 6. | 0. | 5.641827 | 0.577137 | 1.419460 |
| 4. | 6. | 0. | 5.322735 | −0.404160 | 0.475651 |
| 5. | 6. | 0. | 4.366380 | −0.130512 | −0.505728 |
| 6. | 6. | 0. | 3.744550 | 1.124287 | −0.532882 |
| 7. | 6. | 0. | 3.964917 | −1.163405 | −1.548593 |
| 8. | 8. | 0. | 3.507406 | 3.342214 | 0.406398 |
| 9. | 6. | 0. | 2.541702 | −1.719428 | −1.310355 |
| 10. | 8. | 0. | 4.941953 | −2.203038 | −1.557415 |
| 11. | 6. | 0. | 4.972350 | −2.967957 | −2.743440 |
| 12. | 6. | 0. | 2.413844 | −2.556383 | −0.030680 |
| 13. | 6. | 0. | 0.982529 | −3.074547 | 0.231710 |
| 14. | 6. | 0. | 0.974218 | −4.338962 | 1.104497 |
| 15. | 6. | 0. | 0.005925 | −1.981520 | 0.731915 |
| 16. | 6. | 0. | −0.072384 | −1.740070 | 2.258845 |
| 17. | 6. | 0. | −1.229219 | −0.773775 | 2.559899 |
| 18. | 6. | 0. | 1.236164 | −1.253518 | 2.897862 |
| 19. | 6. | 0. | −2.380616 | −1.530572 | 0.444432 |
| 20. | 8. | 0. | −0.807444 | 0.526124 | 2.035550 |
| 21. | 6. | 0. | −2.284679 | 0.811633 | −2.359815 |
| 22. | 6. | 0. | −2.955710 | −0.181599 | −1.391095 |
| 23. | 6. | 0. | −4.414715 | 0.159375 | −0.951022 |
| 24. | 6. | 0. | −4.793935 | −1.153663 | −0.187951 |
| 25. | 6. | 0. | −3.573773 | −2.098715 | −0.381450 |
| 26. | 6. | 0. | −1.094346 | 1.516571 | −1.740460 |
| 27. | 8. | 0. | 0.048150 | 1.402313 | −2.160478 |
| 28. | 8. | 0. | −1.289317 | −2.405607 | 0.239257 |
| 29. | 6. | 0. | −3.868449 | −3.575301 | −0.163152 |
| 30. | 8. | 0. | −5.142808 | 0.277581 | −2.184314 |
| 31. | 6. | 0. | −2.543897 | −1.269218 | 1.941029 |
| 32. | 8. | 0. | −1.423350 | 2.299339 | −0.698486 |
| 33. | 6. | 0. | −1.584367 | 1.598706 | 2.244536 |

| | | | | | |
|---|---|---|---|---|---|
| 34. | 8. | 0. | −2.595434 | 1.597414 | 2.917359 |
| 35. | 6. | 0. | −1.021167 | 2.859787 | 1.598239 |
| 36. | 6. | 0. | −0.360905 | 2.693817 | 0.229291 |
| 37. | 6. | 0. | 0.369303 | 3.966808 | −0.243072 |
| 38. | 6. | 0. | −0.559834 | 5.077889 | −0.728231 |
| 39. | 8. | 0. | 1.347238 | 3.660442 | −1.245961 |
| 40. | 6. | 0. | −3.094593 | −1.656907 | −1.791285 |
| 41. | 8. | 0. | −2.155423 | −0.258961 | −0.190074 |
| 42. | 6. | 0. | −4.590101 | 1.432803 | −0.131058 |
| 43. | 1. | 0. | 5.275866 | 2.595393 | 2.119977 |
| 44. | 1. | 0. | 6.387578 | 0.364742 | 2.181024 |
| 45. | 1. | 0. | 5.816828 | −1.367647 | 0.484858 |
| 46. | 1. | 0. | 3.008225 | 1.349247 | −1.300146 |
| 47. | 1. | 0. | 3.967852 | −0.667303 | −2.534190 |
| 48. | 1. | 0. | 2.862864 | 3.420980 | −0.331489 |
| 49. | 1. | 0. | 1.848279 | −0.870446 | −1.303852 |
| 50. | 1. | 0. | 2.250026 | −2.333314 | −2.173098 |
| 51. | 1. | 0. | 5.121999 | −2.333778 | −3.631094 |
| 52. | 1. | 0. | 5.817289 | −3.655719 | −2.657013 |
| 53. | 1. | 0. | 4.057327 | −3.559247 | −2.894093 |
| 54. | 1. | 0. | 2.784949 | −1.979926 | 0.821683 |
| 55. | 1. | 0. | 3.085982 | −3.417362 | −0.118988 |
| 56. | 1. | 0. | 0.565451 | −3.362370 | −0.742666 |
| 57. | 1. | 0. | 1.458748 | −4.178175 | 2.073778 |
| 58. | 1. | 0. | −0.047878 | −4.685525 | 1.286525 |
| 59. | 1. | 0. | 1.516219 | −5.147061 | 0.602990 |
| 60. | 1. | 0. | 0.255258 | −1.031079 | 0.245907 |
| 61. | 1. | 0. | −0.359197 | −2.689422 | 2.728924 |
| 62. | 1. | 0. | −1.360399 | −0.654909 | 3.640671 |
| 63. | 1. | 0. | 2.003949 | −2.029761 | 2.887944 |
| 64. | 1. | 0. | 1.635959 | −0.375022 | 2.385091 |
| 65. | 1. | 0. | 1.063345 | −0.977351 | 3.944125 |
| 66. | 1. | 0. | −1.914099 | 0.298836 | −3.248458 |
| 67. | 1. | 0. | −3.019497 | 1.554028 | −2.686056 |
| 68. | 1. | 0. | −5.673580 | −1.598927 | −0.663062 |
| 69. | 1. | 0. | −5.039570 | −0.980844 | 0.863356 |
| 70. | 1. | 0. | −2.972626 | −4.179558 | −0.319987 |
| 71. | 1. | 0. | −4.645547 | −3.916213 | −0.855195 |
| 72. | 1. | 0. | −4.229995 | −3.762024 | 0.854780 |
| 73. | 1. | 0. | −6.036937 | 0.575380 | −1.970206 |
| 74. | 1. | 0. | −2.796032 | −2.212425 | 2.438970 |
| 75. | 1. | 0. | −3.340364 | −0.556371 | 2.145916 |
| 76. | 1. | 0. | −1.836960 | 3.584609 | 1.560893 |
| 77. | 1. | 0. | −0.265855 | 3.254485 | 2.289367 |
| 78. | 1. | 0. | 0.364151 | 1.878030 | 0.268265 |
| 79. | 1. | 0. | 0.946327 | 4.327212 | 0.617132 |
| 80. | 1. | 0. | −1.245043 | 5.402190 | 0.061878 |
| 81. | 1. | 0. | −1.155017 | 4.744849 | −1.582727 |
| 82. | 1. | 0. | 0.038962 | 5.937173 | −1.038657 |
| 83. | 1. | 0. | 1.023758 | 2.891007 | −1.757405 |
| 84. | 1. | 0. | −2.143609 | −2.107605 | −2.079289 |
| 85. | 1. | 0. | −3.847345 | −1.811445 | −2.566625 |
| 86. | 1. | 0. | −4.270554 | 2.311216 | −0.698584 |
| 87. | 1. | 0. | −4.032058 | 1.405775 | 0.804932 |
| 88. | 1. | 0. | −5.651157 | 1.560812 | 0.120397 |

| isomer 1-3 | Standard Orientation |
|---|---|

| | | | **(Ångstroms)** | | |
|---|---|---|---|---|---|
| **Center Number** | **Atomic Number** | **Atomic Type** | **X** | Y | Z |
| 1. | 6. | 0. | 4.059949 | 2.000111 | 0.474233 |
| 2. | 6. | 0. | 4.887574 | 1.601696 | 1.534668 |
| 3. | 6. | 0. | 5.393400 | 0.305988 | 1.561084 |
| 4. | 6. | 0. | 5.086959 | −0.608738 | 0.548796 |
| 5. | 6. | 0. | 4.265960 | −0.216527 | −0.513016 |
| 6. | 6. | 0. | 3.760579 | 1.088105 | −0.546678 |
| 7. | 6. | 0. | 3.871650 | −1.189120 | −1.622012 |
| 8. | 8. | 0. | 3.590465 | 3.273938 | 0.477803 |
| 9. | 6. | 0. | 2.464963 | −1.781054 | −1.420902 |
| 10. | 8. | 0. | 4.776119 | −2.281727 | −1.747436 |
| 11. | 6. | 0. | 5.996808 | −1.945097 | −2.381931 |
| 12. | 6. | 0. | 2.320771 | −2.685085 | −0.189059 |
| 13. | 6. | 0. | 0.871361 | −3.166354 | 0.046415 |
| 14. | 6. | 0. | 0.815458 | −4.475100 | 0.848791 |
| 15. | 6. | 0. | −0.064956 | −2.066890 | 0.606683 |
| 16. | 6. | 0. | −0.130759 | −1.900909 | 2.144462 |
| 17. | 6. | 0. | −1.250398 | −0.908868 | 2.497478 |
| 18. | 6. | 0. | 1.195256 | −1.497923 | 2.804526 |
| 19. | 6. | 0. | −2.436948 | −1.520746 | 0.354612 |
| 20. | 8. | 0. | −0.783989 | 0.399859 | 2.032629 |
| 21. | 6. | 0. | −2.285392 | 0.953422 | −2.326930 |
| 22. | 6. | 0. | −2.986964 | −0.063284 | −1.404284 |
| 23. | 6. | 0. | −4.438877 | 0.293734 | −0.931870 |
| 24. | 6. | 0. | −4.844751 | −1.044356 | −0.243591 |
| 25. | 6. | 0. | −3.651991 | −2.009678 | −0.489346 |
| 26. | 6. | 0. | −1.066518 | 1.585055 | −1.681832 |
| 27. | 8. | 0. | 0.068313 | 1.449453 | −2.114232 |
| 28. | 8. | 0. | −1.375435 | −2.418301 | 0.098239 |
| 29. | 6. | 0. | −3.989732 | −3.486316 | −0.346192 |
| 30. | 8. | 0. | −5.275573 | 0.442721 | −2.092352 |
| 31. | 6. | 0. | −2.584212 | −1.327991 | 1.863072 |
| 32. | 8. | 0. | −1.364359 | 2.331066 | −0.605004 |
| 33. | 6. | 0. | −1.521326 | 1.486978 | 2.299086 |
| 34. | 8. | 0. | −2.529031 | 1.488660 | 2.976899 |
| 35. | 6. | 0. | −0.918312 | 2.760304 | 1.714601 |
| 36. | 6. | 0. | −0.281105 | 2.650142 | 0.329407 |
| 37. | 6. | 0. | 0.469419 | 3.929952 | −0.088092 |
| 38. | 6. | 0. | −0.442219 | 5.088100 | −0.489273 |
| 39. | 8. | 0. | 1.416086 | 3.659094 | −1.131592 |
| 40. | 6. | 0. | −3.171123 | −1.509801 | −1.879081 |
| 41. | 8. | 0. | −2.174535 | −0.225772 | −0.217185 |
| 42. | 6. | 0. | −4.568880 | 1.525269 | −0.043379 |
| 43. | 1. | 0. | 5.120765 | 2.320065 | 2.313793 |
| 44. | 1. | 0. | 6.036548 | 0.002274 | 2.382841 |
| 45. | 1. | 0. | 5.480631 | −1.618764 | 0.571954 |
| 46. | 1. | 0. | 3.129154 | 1.401653 | −1.374252 |
| 47. | 1. | 0. | 3.855603 | −0.627939 | −2.571963 |
| 48. | 1. | 0. | 2.956090 | 3.401968 | −0.262242 |
| 49. | 1. | 0. | 1.758593 | −0.943667 | −1.379985 |
| 50. | 1. | 0. | 2.216642 | −2.357793 | −2.320814 |
| 51. | 1. | 0. | 6.561613 | −1.189287 | −1.820117 |
| 52. | 1. | 0. | 6.589727 | −2.861226 | −2.440557 |
| 53. | 1. | 0. | 5.827092 | −1.566239 | −3.401683 |
| 54. | 1. | 0. | 2.707064 | −2.169440 | 0.694591 |
| 55. | 1. | 0. | 2.962733 | −3.560935 | −0.332152 |
| 56. | 1. | 0. | 0.445394 | −3.385303 | −0.941781 |

| | | | | | |
|---|---|---|---|---|---|
| 57. | 1. | 0. | 1.302683 | −4.385983 | 1.826093 |
| 58. | 1. | 0. | −0.219138 | −4.793315 | 1.010996 |
| 59. | 1. | 0. | 1.328980 | −5.273462 | 0.303763 |
| 60. | 1. | 0. | 0.216233 | −1.101476 | 0.169977 |
| 61. | 1. | 0. | −0.452742 | −2.861555 | 2.566531 |
| 62. | 1. | 0. | −1.373254 | −0.835927 | 3.583206 |
| 63. | 1. | 0. | 1.932614 | −2.301437 | 2.752281 |
| 64. | 1. | 0. | 1.629064 | −0.609652 | 2.338077 |
| 65. | 1. | 0. | 1.033257 | −1.270987 | 3.864352 |
| 66. | 1. | 0. | −1.939857 | 0.476924 | −3.245405 |
| 67. | 1. | 0. | −2.980473 | 1.750697 | −2.611690 |
| 68. | 1. | 0. | −5.737921 | −1.429384 | −0.742736 |
| 69. | 1. | 0. | −5.087605 | −0.917437 | 0.814520 |
| 70. | 1. | 0. | −3.113226 | −4.108388 | −0.538337 |
| 71. | 1. | 0. | −4.779101 | −3.766615 | −1.051223 |
| 72. | 1. | 0. | −4.354242 | −3.714630 | 0.662144 |
| 73. | 1. | 0. | −5.295861 | 1.379487 | −2.328032 |
| 74. | 1. | 0. | −2.866050 | −2.285292 | 2.316207 |
| 75. | 1. | 0. | −3.355067 | −0.599611 | 2.107021 |
| 76. | 1. | 0. | −1.707063 | 3.515132 | 1.730305 |
| 77. | 1. | 0. | −0.139915 | 3.088183 | 2.415025 |
| 78. | 1. | 0. | 0.424229 | 1.816413 | 0.313900 |
| 79. | 1. | 0. | 1.073710 | 4.227565 | 0.777372 |
| 80. | 1. | 0. | −1.099094 | 5.386931 | 0.334200 |
| 81. | 1. | 0. | −1.066003 | 4.815984 | −1.344905 |
| 82. | 1. | 0. | 0.171141 | 5.947670 | −0.768971 |
| 83. | 1. | 0. | 1.080155 | 2.905165 | −1.657495 |
| 84. | 1. | 0. | −2.237489 | −1.972992 | −2.202184 |
| 85. | 1. | 0. | −3.936826 | −1.601002 | −2.652108 |
| 86. | 1. | 0. | −4.240170 | 2.430917 | −0.568469 |
| 87. | 1. | 0. | −3.977383 | 1.444213 | 0.868119 |
| 88. | 1. | 0. | −5.619587 | 1.656457 | 0.235001 |

| **Isomer 1-4** | | | **Standard Orientation (Ångstroms)** | | |
|---|---|---|---|---|---|
| **Center Number** | **Atomic Number** | **Atomic Type** | **X** | Y | Z |
| 1. | 6. | 0. | 4.045504 | 1.994768 | 0.481904 |
| 2. | 6. | 0. | 4.878316 | 1.593618 | 1.537465 |
| 3. | 6. | 0. | 5.387807 | 0.299300 | 1.556404 |
| 4. | 6. | 0. | 5.080148 | −0.611805 | 0.541180 |
| 5. | 6. | 0. | 4.253847 | −0.217117 | −0.515602 |
| 6. | 6. | 0. | 3.744381 | 1.086147 | −0.541759 |
| 7. | 6. | 0. | 3.858085 | −1.186388 | −1.626973 |
| 8. | 8. | 0. | 3.573293 | 3.266914 | 0.493373 |
| 9. | 6. | 0. | 2.453976 | −1.783217 | −1.422328 |
| 10. | 8. | 0. | 4.764693 | −2.276295 | −1.760372 |
| 11. | 6. | 0. | 5.982584 | −1.933735 | −2.397205 |
| 12. | 6. | 0. | 2.317275 | −2.690675 | −0.192050 |
| 13. | 6. | 0. | 0.870079 | −3.175367 | 0.049621 |
| 14. | 6. | 0. | 0.820326 | −4.481624 | 0.856474 |
| 15. | 6. | 0. | −0.065791 | −2.075828 | 0.609597 |
| 16. | 6. | 0. | −0.129758 | −1.904513 | 2.146401 |
| 17. | 6. | 0. | −1.246028 | −0.907308 | 2.496227 |
| 18. | 6. | 0. | 1.197789 | −1.501603 | 2.803478 |
| 19. | 6. | 0. | −2.434590 | −1.523191 | 0.356851 |
| 20. | 8. | 0. | −0.778201 | 0.397195 | 2.025538 |
| 21. | 6. | 0. | −2.284527 | 0.958516 | −2.328848 |

| | | | | | |
|---|---|---|---|---|---|
| 22. | 6. | 0. | −2.974532 | −0.064257 | −1.406716 |
| 23. | 6. | 0. | −4.424476 | 0.302426 | −0.935330 |
| 24. | 6. | 0. | −4.841520 | −1.040908 | −0.250090 |
| 25. | 6. | 0. | −3.650204 | −2.011207 | −0.488581 |
| 26. | 6. | 0. | −1.071602 | 1.603581 | −1.687039 |
| 27. | 8. | 0. | 0.061042 | 1.493307 | −2.134147 |
| 28. | 8. | 0. | −1.378880 | −2.429375 | 0.106137 |
| 29. | 6. | 0. | −3.990095 | −3.487275 | −0.342875 |
| 30. | 8. | 0. | −5.237540 | 0.612921 | −2.075223 |
| 31. | 6. | 0. | −2.582920 | −1.322884 | 1.864054 |
| 32. | 8. | 0. | −1.368428 | 2.327272 | −0.595677 |
| 33. | 6. | 0. | −1.506419 | 1.487432 | 2.310417 |
| 34. | 8. | 0. | −2.499794 | 1.487049 | 3.008470 |
| 35. | 6. | 0. | −0.912217 | 2.761474 | 1.719554 |
| 36. | 6. | 0. | −0.282081 | 2.655440 | 0.330448 |
| 37. | 6. | 0. | 0.456339 | 3.942826 | −0.088716 |
| 38. | 6. | 0. | −0.467225 | 5.088050 | −0.499559 |
| 39. | 8. | 0. | 1.413723 | 3.678248 | −1.123678 |
| 40. | 6. | 0. | −3.151202 | −1.517712 | −1.874709 |
| 41. | 8. | 0. | −2.161881 | −0.235135 | −0.222758 |
| 42. | 6. | 0. | −4.538789 | 1.526040 | −0.037018 |
| 43. | 1. | 0. | 5.112621 | 2.309352 | 2.318677 |
| 44. | 1. | 0. | 6.035026 | −0.006151 | 2.374318 |
| 45. | 1. | 0. | 5.476845 | −1.620783 | 0.558312 |
| 46. | 1. | 0. | 3.108205 | 1.401600 | −1.364970 |
| 47. | 1. | 0. | 3.836492 | −0.621645 | −2.574666 |
| 48. | 1. | 0. | 2.940265 | 3.400648 | −0.247366 |
| 49. | 1. | 0. | 1.745640 | −0.947763 | −1.376673 |
| 50. | 1. | 0. | 2.204054 | −2.358446 | −2.322836 |
| 51. | 1. | 0. | 6.547557 | −1.179664 | −1.833314 |
| 52. | 1. | 0. | 6.577228 | −2.848289 | −2.462580 |
| 53. | 1. | 0. | 5.808577 | −1.549913 | −3.414363 |
| 54. | 1. | 0. | 2.706576 | −2.176423 | 0.691086 |
| 55. | 1. | 0. | 2.960195 | −3.564977 | −0.340155 |
| 56. | 1. | 0. | 0.441726 | −3.398585 | −0.936718 |
| 57. | 1. | 0. | 1.309957 | −4.387594 | 1.832040 |
| 58. | 1. | 0. | −0.212767 | −4.802634 | 1.023047 |
| 59. | 1. | 0. | 1.334775 | −5.280259 | 0.312744 |
| 60. | 1. | 0. | 0.212122 | −1.111537 | 0.168964 |
| 61. | 1. | 0. | −0.453483 | −2.862951 | 2.572237 |
| 62. | 1. | 0. | −1.369223 | −0.831098 | 3.581579 |
| 63. | 1. | 0. | 1.934352 | −2.305862 | 2.751678 |
| 64. | 1. | 0. | 1.631387 | −0.614551 | 2.334633 |
| 65. | 1. | 0. | 1.037520 | −1.272470 | 3.863043 |
| 66. | 1. | 0. | −1.929648 | 0.485860 | −3.246243 |
| 67. | 1. | 0. | −3.006625 | 1.732732 | −2.608140 |
| 68. | 1. | 0. | −5.741071 | −1.434854 | −0.736434 |
| 69. | 1. | 0. | −5.087826 | −0.916308 | 0.807476 |
| 70. | 1. | 0. | −3.113503 | −4.110686 | −0.529567 |
| 71. | 1. | 0. | −4.778024 | −3.771168 | −1.048657 |
| 72. | 1. | 0. | −4.358105 | −3.710878 | 0.664958 |
| 73. | 1. | 0. | −5.337936 | −0.182759 | −2.613399 |
| 74. | 1. | 0. | −2.870648 | −2.276295 | 2.321664 |
| 75. | 1. | 0. | −3.349140 | −0.588148 | 2.104362 |
| 76. | 1. | 0. | −1.704558 | 3.512510 | 1.739271 |
| 77. | 1. | 0. | −0.132010 | 3.093801 | 2.415899 |
| 78. | 1. | 0. | 0.430712 | 1.827985 | 0.310572 |
| 79. | 1. | 0. | 1.052376 | 4.251369 | 0.778509 |

| | | | | | |
|---|---|---|---|---|---|
| 80. | 1. | 0. | −1.135561 | 5.378499 | 0.317627 |
| 81. | 1. | 0. | −1.080658 | 4.807216 | −1.359802 |
| 82. | 1. | 0. | 0.136919 | 5.955557 | −0.774814 |
| 83. | 1. | 0. | 1.073449 | 2.937671 | −1.666058 |
| 84. | 1. | 0. | −2.212502 | −1.980180 | −2.182646 |
| 85. | 1. | 0. | −3.893045 | −1.648375 | −2.669848 |
| 86. | 1. | 0. | −4.203951 | 2.425124 | −0.561285 |
| 87. | 1. | 0. | −3.953543 | 1.419101 | 0.874992 |
| 88. | 1. | 0. | −5.589840 | 1.667391 | 0.231460 |

**Table S1.4.2.a.** Gibbs free energies[a] and equilibrium populations[b] of low−energy conformers of isomer 2.

| Conformers | In MeOH | |
|---|---|---|
| | **Δ*G*** | ***P* (%)** |
| isomer 2-1 | 0 | 71.59 |
| isomer 2-2 | 1.32 | 14.48 |
| isomer 2-3 | 1.96 | 6.82 |
| isomer 2-4 | 1.91 | 7.11 |

[a]B3LYP/6−31+G(d, p), in kcal/mol. [b]From Δ*G* values at 298.15 K.

**Table S1.4.2.b.** Cartesian coordinates for the low-energy reoptimized MMFF conformers of isomer 2 at B3LYP/6−31G (d, p) level of theory in $CH_3OH$.

| **Isomer 2-1** | | | **Standard Orientation (Ångstroms)** | | |
|---|---|---|---|---|---|
| **Center Number** | **Atomic Number** | **Atomic Type** | **X** | Y | Z |
| 1. | 6. | 0. | 2.129215 | 3.024983 | −0.851007 |
| 2. | 6. | 0. | 2.831412 | 3.787841 | 0.092536 |
| 3. | 6. | 0. | 4.006555 | 3.284867 | 0.640400 |
| 4. | 6. | 0. | 4.494588 | 2.029562 | 0.264858 |
| 5. | 6. | 0. | 3.806649 | 1.272412 | −0.687681 |
| 6. | 6. | 0. | 2.624440 | 1.777722 | −1.246564 |
| 7. | 6. | 0. | 4.294552 | −0.115220 | −1.102880 |
| 8. | 8. | 0. | 0.976852 | 3.555640 | −1.349550 |
| 9. | 6. | 0. | 3.423699 | −1.239974 | −0.518422 |
| 10. | 8. | 0. | 5.639817 | −0.368545 | −0.714916 |
| 11. | 6. | 0. | 6.604615 | 0.222535 | −1.567906 |
| 12. | 6. | 0. | 3.442103 | −1.368197 | 1.014004 |
| 13. | 6. | 0. | 2.267975 | −2.229085 | 1.539495 |
| 14. | 6. | 0. | 2.557119 | −2.882266 | 2.898328 |
| 15. | 6. | 0. | 0.925060 | −1.462965 | 1.476230 |
| 16. | 6. | 0. | 0.474113 | −0.665439 | 2.718529 |
| 17. | 6. | 0. | −0.940868 | −0.100679 | 2.501468 |
| 18. | 6. | 0. | 1.437569 | 0.450596 | 3.148583 |
| 19. | 6. | 0. | −1.318465 | −1.916654 | 0.747948 |
| 20. | 8. | 0. | −0.859851 | 1.033491 | 1.580588 |
| 21. | 6. | 0. | −1.832740 | −0.482648 | −2.591226 |
| 22. | 6. | 0. | −1.812511 | −1.582636 | −1.528444 |
| 23. | 6. | 0. | −1.077854 | −2.897000 | −1.989651 |
| 24. | 6. | 0. | −1.456988 | −3.866641 | −0.822160 |
| 25. | 6. | 0. | −2.282587 | −2.997645 | 0.159005 |
| 26. | 6. | 0. | −1.761466 | 0.932771 | −2.051208 |
| 27. | 8. | 0. | −0.907168 | 1.733192 | −2.393662 |
| 28. | 8. | 0. | −0.082189 | −2.470051 | 1.153091 |
| 29. | 6. | 0. | −3.090019 | −3.799263 | 1.172190 |
| 30. | 8. | 0. | 0.317982 | −2.670264 | −2.104388 |

| | | | | | |
|---|---|---|---|---|---|
| 31. | 6. | 0. | −1.906888 | −1.133210 | 1.927267 |
| 32. | 8. | 0. | −2.765290 | 1.216635 | −1.212361 |
| 33. | 6. | 0. | −1.909706 | 1.862687 | 1.571154 |
| 34. | 8. | 0. | −2.813114 | 1.818782 | 2.394480 |
| 35. | 6. | 0. | −1.857281 | 2.843329 | 0.420410 |
| 36. | 6. | 0. | −2.888141 | 2.571037 | −0.696758 |
| 37. | 6. | 0. | −4.386735 | 2.726148 | −0.350255 |
| 38. | 6. | 0. | −4.723602 | 4.131375 | 0.157987 |
| 39. | 8. | 0. | −4.867973 | 1.719519 | 0.519736 |
| 40. | 6. | 0. | −3.075492 | −2.107526 | −0.841792 |
| 41. | 8. | 0. | −1.068541 | −1.065387 | −0.395138 |
| 42. | 6. | 0. | −1.525531 | −3.418359 | −3.353227 |
| 43. | 1. | 0. | 2.438946 | 4.758270 | 0.378101 |
| 44. | 1. | 0. | 4.548090 | 3.875044 | 1.374735 |
| 45. | 1. | 0. | 5.401931 | 1.631406 | 0.704581 |
| 46. | 1. | 0. | 2.076569 | 1.203040 | −1.990651 |
| 47. | 1. | 0. | 4.218787 | −0.185680 | −2.201355 |
| 48. | 1. | 0. | 0.484988 | 2.896970 | −1.872936 |
| 49. | 1. | 0. | 2.403480 | −1.058264 | −0.876007 |
| 50. | 1. | 0. | 3.745937 | −2.187386 | −0.966713 |
| 51. | 1. | 0. | 6.525883 | 1.317761 | −1.588008 |
| 52. | 1. | 0. | 7.587392 | −0.055668 | −1.179507 |
| 53. | 1. | 0. | 6.510780 | −0.152607 | −2.598469 |
| 54. | 1. | 0. | 3.420288 | −0.374576 | 1.471725 |
| 55. | 1. | 0. | 4.391630 | −1.821328 | 1.317790 |
| 56. | 1. | 0. | 2.134677 | −3.051105 | 0.823285 |
| 57. | 1. | 0. | 1.703164 | −3.477297 | 3.237814 |
| 58. | 1. | 0. | 3.417816 | −3.553717 | 2.816852 |
| 59. | 1. | 0. | 2.792123 | −2.148757 | 3.676435 |
| 60. | 1. | 0. | 0.966009 | −0.761655 | 0.636271 |
| 61. | 1. | 0. | 0.368190 | −1.378865 | 3.546807 |
| 62. | 1. | 0. | −1.333492 | 0.284112 | 3.446473 |
| 63. | 1. | 0. | 1.637417 | 1.146408 | 2.329723 |
| 64. | 1. | 0. | 0.999832 | 1.024443 | 3.972959 |
| 65. | 1. | 0. | 2.391857 | 0.052008 | 3.498067 |
| 66. | 1. | 0. | −0.956599 | −0.595300 | −3.232809 |
| 67. | 1. | 0. | −2.732007 | −0.574592 | −3.209947 |
| 68. | 1. | 0. | −0.564456 | −4.293724 | −0.363445 |
| 69. | 1. | 0. | −2.080281 | −4.691620 | −1.184098 |
| 70. | 1. | 0. | −2.431236 | −4.326492 | 1.870663 |
| 71. | 1. | 0. | −3.769745 | −3.168589 | 1.752328 |
| 72. | 1. | 0. | −3.697118 | −4.550997 | 0.657590 |
| 73. | 1. | 0. | 0.600214 | −2.321732 | −1.245939 |
| 74. | 1. | 0. | −2.851709 | −0.654873 | 1.663045 |
| 75. | 1. | 0. | −2.116666 | −1.856752 | 2.721518 |
| 76. | 1. | 0. | −0.858096 | 2.860010 | −0.012329 |
| 77. | 1. | 0. | −2.061971 | 3.839479 | 0.824608 |
| 78. | 1. | 0. | −2.648479 | 3.261188 | −1.512708 |
| 79. | 1. | 0. | −4.916851 | 2.560050 | −1.296339 |
| 80. | 1. | 0. | −4.366795 | 4.911380 | −0.523940 |
| 81. | 1. | 0. | −4.285625 | 4.309521 | 1.145641 |
| 82. | 1. | 0. | −5.807869 | 4.227061 | 0.253722 |
| 83. | 1. | 0. | −4.420337 | 1.834645 | 1.376162 |
| 84. | 1. | 0. | −3.664464 | −1.309449 | −0.383027 |
| 85. | 1. | 0. | −3.723318 | −2.699289 | −1.495744 |
| 86. | 1. | 0. | −1.229754 | −2.733762 | −4.152796 |
| 87. | 1. | 0. | −2.608252 | −3.566730 | −3.395902 |
| 88. | 1. | 0. | −1.041818 | −4.380842 | −3.544522 |

| **Isomer 2-2** | | | **Standard Orientation (Ångstroms)** | | |
|---|---|---|---|---|---|
| **Center Number** | **Atomic Number** | **Atomic Type** | **X** | Y | Z |
| 1. | 6. | 0. | 1.964059 | 3.186829 | −0.912486 |
| 2. | 6. | 0. | 2.687095 | 4.089483 | −0.120965 |
| 3. | 6. | 0. | 3.946823 | 3.730279 | 0.345284 |
| 4. | 6. | 0. | 4.501226 | 2.483541 | 0.040441 |
| 5. | 6. | 0. | 3.782805 | 1.579789 | −0.746576 |
| 6. | 6. | 0. | 2.512592 | 1.938064 | −1.222966 |
| 7. | 6. | 0. | 4.349594 | 0.210587 | −1.103316 |
| 8. | 8. | 0. | 0.736452 | 3.586026 | −1.351926 |
| 9. | 6. | 0. | 3.500713 | −0.944902 | −0.529780 |
| 10. | 8. | 0. | 5.698871 | 0.144236 | −0.650381 |
| 11. | 6. | 0. | 6.487418 | −0.831090 | −1.299754 |
| 12. | 6. | 0. | 3.522115 | −1.050859 | 1.003936 |
| 13. | 6. | 0. | 2.416682 | −1.985521 | 1.549788 |
| 14. | 6. | 0. | 2.759239 | −2.590324 | 2.918888 |
| 15. | 6. | 0. | 1.019722 | −1.324246 | 1.481942 |
| 16. | 6. | 0. | 0.519526 | −0.540253 | 2.714503 |
| 17. | 6. | 0. | −0.939064 | −0.095163 | 2.506131 |
| 18. | 6. | 0. | 1.397122 | 0.657987 | 3.105953 |
| 19. | 6. | 0. | −1.187688 | −1.961722 | 0.784649 |
| 20. | 8. | 0. | −0.955279 | 1.027379 | 1.569356 |
| 21. | 6. | 0. | −1.852859 | −0.615470 | −2.566286 |
| 22. | 6. | 0. | −1.728096 | −1.697528 | −1.491386 |
| 23. | 6. | 0. | −0.893492 | −2.952973 | −1.944561 |
| 24. | 6. | 0. | −1.179844 | −3.934728 | −0.761153 |
| 25. | 6. | 0. | −2.065559 | −3.124476 | 0.218113 |
| 26. | 6. | 0. | −1.861734 | 0.806484 | −2.038586 |
| 27. | 8. | 0. | −1.044188 | 1.646396 | −2.374956 |
| 28. | 8. | 0. | 0.091434 | −2.410583 | 1.185034 |
| 29. | 6. | 0. | −2.793227 | −3.979303 | 1.247906 |
| 30. | 8. | 0. | 0.477994 | −2.613909 | −2.078775 |
| 31. | 6. | 0. | −1.825456 | −1.211126 | 1.958418 |
| 32. | 8. | 0. | −2.890652 | 1.045279 | −1.215863 |
| 33. | 6. | 0. | −2.068864 | 1.768629 | 1.550651 |
| 34. | 8. | 0. | −2.970114 | 1.654518 | 2.369423 |
| 35. | 6. | 0. | −2.084783 | 2.746521 | 0.397100 |
| 36. | 6. | 0. | −3.092610 | 2.398207 | −0.719782 |
| 37. | 6. | 0. | −4.598978 | 2.471831 | −0.379311 |
| 38. | 6. | 0. | −5.016976 | 3.861922 | 0.111067 |
| 39. | 8. | 0. | −5.025021 | 1.450736 | 0.501932 |
| 40. | 6. | 0. | −2.937681 | −2.314150 | −0.785150 |
| 41. | 8. | 0. | −1.016677 | −1.107995 | −0.371835 |
| 42. | 6. | 0. | −1.310511 | −3.527559 | −3.296492 |
| 43. | 1. | 0. | 2.248239 | 5.055323 | 0.106872 |
| 44. | 1. | 0. | 4.508897 | 4.431846 | 0.955799 |
| 45. | 1. | 0. | 5.486115 | 2.210163 | 0.396710 |
| 46. | 1. | 0. | 1.942182 | 1.255148 | −1.848803 |
| 47. | 1. | 0. | 4.339806 | 0.117321 | −2.203128 |
| 48. | 1. | 0. | 0.285760 | 2.872756 | −1.839204 |
| 49. | 1. | 0. | 2.477837 | −0.800683 | −0.895857 |
| 50. | 1. | 0. | 3.836872 | −1.894579 | −0.964976 |
| 51. | 1. | 0. | 6.153022 | −1.857524 | −1.090322 |
| 52. | 1. | 0. | 6.493160 | −0.686672 | −2.391160 |
| 53. | 1. | 0. | 7.507204 | −0.716505 | −0.924291 |
| 54. | 1. | 0. | 3.432290 | −0.052308 | 1.442343 |
| 55. | 1. | 0. | 4.501650 | −1.427441 | 1.318099 |

| | | | | | |
|---|---|---|---|---|---|
| 56. | 1. | 0. | 2.341245 | −2.828650 | 0.849696 |
| 57. | 1. | 0. | 3.667757 | −3.196700 | 2.845759 |
| 58. | 1. | 0. | 2.940706 | −1.827164 | 3.682657 |
| 59. | 1. | 0. | 1.953630 | −3.241632 | 3.272847 |
| 60. | 1. | 0. | 1.003006 | −0.638046 | 0.628844 |
| 61. | 1. | 0. | 0.482450 | −1.242281 | 3.558013 |
| 62. | 1. | 0. | −1.351449 | 0.271314 | 3.450134 |
| 63. | 1. | 0. | 1.530109 | 1.346632 | 2.267726 |
| 64. | 1. | 0. | 0.926257 | 1.215880 | 3.922934 |
| 65. | 1. | 0. | 2.384446 | 0.344297 | 3.450533 |
| 66. | 1. | 0. | −0.989140 | −0.675078 | −3.231292 |
| 67. | 1. | 0. | −2.761387 | −0.776657 | −3.156144 |
| 68. | 1. | 0. | −0.250542 | −4.278772 | −0.305955 |
| 69. | 1. | 0. | −1.735507 | −4.813599 | −1.105823 |
| 70. | 1. | 0. | −2.084209 | −4.450235 | 1.937468 |
| 71. | 1. | 0. | −3.510933 | −3.401379 | 1.837480 |
| 72. | 1. | 0. | −3.348266 | −4.778764 | 0.746578 |
| 73. | 1. | 0. | 0.735333 | −2.214851 | −1.234636 |
| 74. | 1. | 0. | −2.807999 | −0.813360 | 1.698677 |
| 75. | 1. | 0. | −1.969023 | −1.937150 | 2.765079 |
| 76. | 1. | 0. | −1.087392 | 2.832705 | −0.031770 |
| 77. | 1. | 0. | −2.360270 | 3.726660 | 0.798152 |
| 78. | 1. | 0. | −2.889769 | 3.088913 | −1.545205 |
| 79. | 1. | 0. | −5.115873 | 2.264545 | −1.324592 |
| 80. | 1. | 0. | −4.704034 | 4.653332 | −0.579241 |
| 81. | 1. | 0. | −4.592524 | 4.076307 | 1.097367 |
| 82. | 1. | 0. | −6.105157 | 3.895871 | 0.203927 |
| 83. | 1. | 0. | −4.577293 | 1.592978 | 1.354340 |
| 84. | 1. | 0. | −3.584678 | −1.560246 | −0.329936 |
| 85. | 1. | 0. | −3.541801 | −2.964795 | −1.424822 |
| 86. | 1. | 0. | −1.080179 | −2.831696 | −4.107787 |
| 87. | 1. | 0. | −2.377697 | −3.764846 | −3.325390 |
| 88. | 1. | 0. | −0.751253 | −4.449604 | −3.481085 |

| **Isomer 2-3** | | | **Standard Orientation (Ångstroms)** | | |
|---|---|---|---|---|---|
| **Center Number** | **Atomic Number** | **Atomic Type** | **X** | Y | Z |
| 1. | 6. | 0. | 3.717850 | −2.449443 | −1.533096 |
| 2. | 6. | 0. | 4.258587 | −3.488371 | −0.765518 |
| 3. | 6. | 0. | 5.103276 | −3.181570 | 0.300257 |
| 4. | 6. | 0. | 5.403402 | −1.853728 | 0.609969 |
| 5. | 6. | 0. | 4.856542 | −0.813811 | −0.151670 |
| 6. | 6. | 0. | 4.026152 | −1.121118 | −1.233958 |
| 7. | 6. | 0. | 5.109362 | 0.649150 | 0.197840 |
| 8. | 8. | 0. | 2.868893 | −2.672117 | −2.584170 |
| 9. | 6. | 0. | 3.845657 | 1.342038 | 0.732363 |
| 10. | 8. | 0. | 5.509359 | 1.406855 | −0.942127 |
| 11. | 6. | 0. | 6.794882 | 1.076381 | −1.434636 |
| 12. | 6. | 0. | 3.356772 | 0.781618 | 2.078160 |
| 13. | 6. | 0. | 1.920622 | 1.171365 | 2.502888 |
| 14. | 6. | 0. | 1.727934 | 2.692599 | 2.599196 |
| 15. | 6. | 0. | 0.781520 | 0.512071 | 1.692010 |
| 16. | 6. | 0. | 0.867586 | −1.023483 | 1.568200 |
| 17. | 6. | 0. | −0.231651 | −1.560740 | 0.643495 |
| 18. | 6. | 0. | 0.859871 | −1.742012 | 2.923825 |
| 19. | 6. | 0. | −0.362952 | 0.759871 | −0.421997 |
| 20. | 8. | 0. | −1.516450 | −1.498594 | 1.335548 |

| | | | | | |
|---|---|---|---|---|---|
| 21. | 6. | 0. | −3.745475 | 2.182535 | −0.125561 |
| 22. | 6. | 0. | −2.300224 | 2.091472 | −0.615352 |
| 23. | 6. | 0. | −1.514943 | 3.451837 | −0.534554 |
| 24. | 6. | 0. | −0.223951 | 3.087546 | −1.341716 |
| 25. | 6. | 0. | −0.408095 | 1.599598 | −1.738433 |
| 26. | 6. | 0. | −4.318312 | 0.944218 | 0.540637 |
| 27. | 8. | 0. | −4.843577 | 0.956206 | 1.632424 |
| 28. | 8. | 0. | 0.743001 | 1.126984 | 0.377712 |
| 29. | 6. | 0. | 0.508705 | 1.139725 | −2.864505 |
| 30. | 8. | 0. | −1.266087 | 3.789729 | 0.819688 |
| 31. | 6. | 0. | −0.327035 | −0.758209 | −0.651431 |
| 32. | 8. | 0. | −4.226446 | −0.165940 | −0.230442 |
| 33. | 6. | 0. | −2.477957 | −2.313899 | 0.882162 |
| 34. | 8. | 0. | −2.279017 | −3.218380 | 0.081871 |
| 35. | 6. | 0. | −3.843710 | −1.963618 | 1.422100 |
| 36. | 6. | 0. | −4.768735 | −1.395296 | 0.325706 |
| 37. | 6. | 0. | −4.985155 | −2.327941 | −0.892201 |
| 38. | 6. | 0. | −6.276770 | −1.989089 | −1.625402 |
| 39. | 8. | 0. | −5.047359 | −3.687076 | −0.490552 |
| 40. | 6. | 0. | −1.937817 | 1.562330 | −2.003505 |
| 41. | 8. | 0. | −1.580946 | 1.167074 | 0.251819 |
| 42. | 6. | 0. | −2.253841 | 4.648076 | −1.131488 |
| 43. | 1. | 0. | 4.022064 | −4.523571 | −1.002107 |
| 44. | 1. | 0. | 5.529231 | −3.986783 | 0.891784 |
| 45. | 1. | 0. | 6.060772 | −1.624447 | 1.443993 |
| 46. | 1. | 0. | 3.617686 | −0.330932 | −1.854700 |
| 47. | 1. | 0. | 5.905205 | 0.692111 | 0.962174 |
| 48. | 1. | 0. | 2.737333 | −3.623574 | −2.690364 |
| 49. | 1. | 0. | 4.074309 | 2.409429 | 0.820843 |
| 50. | 1. | 0. | 3.060598 | 1.249650 | −0.021501 |
| 51. | 1. | 0. | 6.996615 | 1.749350 | −2.271359 |
| 52. | 1. | 0. | 7.571072 | 1.222125 | −0.667180 |
| 53. | 1. | 0. | 6.850567 | 0.038653 | −1.790974 |
| 54. | 1. | 0. | 4.038650 | 1.117370 | 2.871477 |
| 55. | 1. | 0. | 3.439268 | −0.310902 | 2.067905 |
| 56. | 1. | 0. | 1.792241 | 0.774976 | 3.519285 |
| 57. | 1. | 0. | 0.747786 | 2.940489 | 3.021486 |
| 58. | 1. | 0. | 2.487923 | 3.133254 | 3.253115 |
| 59. | 1. | 0. | 1.797470 | 3.177296 | 1.623379 |
| 60. | 1. | 0. | −0.162675 | 0.756880 | 2.198771 |
| 61. | 1. | 0. | 1.802683 | −1.262893 | 1.046100 |
| 62. | 1. | 0. | −0.040740 | −2.611638 | 0.412439 |
| 63. | 1. | 0. | −0.017078 | −1.461836 | 3.514752 |
| 64. | 1. | 0. | 0.829728 | −2.827270 | 2.779706 |
| 65. | 1. | 0. | 1.754883 | −1.514098 | 3.508060 |
| 66. | 1. | 0. | −3.814477 | 2.968581 | 0.629247 |
| 67. | 1. | 0. | −4.396453 | 2.457792 | −0.963363 |
| 68. | 1. | 0. | 0.670986 | 3.259338 | −0.743890 |
| 69. | 1. | 0. | −0.146006 | 3.693675 | −2.250868 |
| 70. | 1. | 0. | 1.557113 | 1.154749 | −2.548788 |
| 71. | 1. | 0. | 0.272929 | 0.129584 | −3.211228 |
| 72. | 1. | 0. | 0.413677 | 1.813356 | −3.722374 |
| 73. | 1. | 0. | −0.962150 | 2.971412 | 1.241912 |
| 74. | 1. | 0. | −1.189394 | −1.092396 | −1.231181 |
| 75. | 1. | 0. | 0.568527 | −0.985986 | −1.238312 |
| 76. | 1. | 0. | −3.767561 | −1.243731 | 2.236020 |
| 77. | 1. | 0. | −4.314959 | −2.879567 | 1.788163 |
| 78. | 1. | 0. | −5.722962 | −1.160082 | 0.806384 |

| | | | | | |
|---|---|---|---|---|---|
| 79. | 1. | 0. | −4.133755 | −2.169265 | −1.569235 |
| 80. | 1. | 0. | −6.294384 | −0.933610 | −1.912397 |
| 81. | 1. | 0. | −7.139146 | −2.193049 | −0.982231 |
| 82. | 1. | 0. | −6.370726 | −2.601326 | −2.525771 |
| 83. | 1. | 0. | −4.124166 | −3.952646 | −0.336084 |
| 84. | 1. | 0. | −2.346179 | 0.567488 | −2.194970 |
| 85. | 1. | 0. | −2.234029 | 2.230748 | −2.817606 |
| 86. | 1. | 0. | −3.148894 | 4.887744 | −0.551430 |
| 87. | 1. | 0. | −2.540833 | 4.472785 | −2.172343 |
| 88. | 1. | 0. | −1.596301 | 5.522235 | −1.103640 |

| **isomer 2-4** | | | **Standard Orientation (Ångstroms)** | | |
|---|---|---|---|---|---|
| **Center Number** | **Atomic Number** | **Atomic Type** | **X** | Y | Z |
| 1. | 6. | 0. | 5.069767 | −2.394771 | −1.517908 |
| 2. | 6. | 0. | 5.649149 | −3.242518 | −0.565537 |
| 3. | 6. | 0. | 6.055463 | −2.714337 | 0.655522 |
| 4. | 6. | 0. | 5.890723 | −1.354847 | 0.938016 |
| 5. | 6. | 0. | 5.299737 | −0.509730 | −0.005595 |
| 6. | 6. | 0. | 4.893003 | −1.038368 | −1.237336 |
| 7. | 6. | 0. | 5.062149 | 0.966320 | 0.290840 |
| 8. | 8. | 0. | 4.696440 | −2.953157 | −2.709203 |
| 9. | 6. | 0. | 3.603870 | 1.274849 | 0.667495 |
| 10. | 8. | 0. | 5.349697 | 1.787712 | −0.840165 |
| 11. | 6. | 0. | 6.729219 | 1.881967 | −1.147302 |
| 12. | 6. | 0. | 3.161838 | 0.631371 | 1.988896 |
| 13. | 6. | 0. | 1.718324 | 0.928630 | 2.459822 |
| 14. | 6. | 0. | 1.465750 | 2.427376 | 2.690949 |
| 15. | 6. | 0. | 0.591313 | 0.301448 | 1.613569 |
| 16. | 6. | 0. | 0.688373 | −1.222644 | 1.399831 |
| 17. | 6. | 0. | −0.434775 | −1.704061 | 0.471477 |
| 18. | 6. | 0. | 0.714258 | −2.022685 | 2.708886 |
| 19. | 6. | 0. | −0.571415 | 0.674422 | −0.467458 |
| 20. | 8. | 0. | −1.701446 | −1.665403 | 1.195682 |
| 21. | 6. | 0. | −3.926335 | 2.126829 | −0.041774 |
| 22. | 6. | 0. | −2.491102 | 2.040814 | −0.561945 |
| 23. | 6. | 0. | −1.682049 | 3.383050 | −0.425451 |
| 24. | 6. | 0. | −0.411198 | 3.041782 | −1.273845 |
| 25. | 6. | 0. | −0.625432 | 1.578904 | −1.740286 |
| 26. | 6. | 0. | −4.497959 | 0.865619 | 0.582105 |
| 27. | 8. | 0. | −4.993216 | 0.832513 | 1.687229 |
| 28. | 8. | 0. | 0.549591 | 0.987340 | 0.333988 |
| 29. | 6. | 0. | 0.266176 | 1.161119 | −2.902563 |
| 30. | 8. | 0. | −1.403023 | 3.647146 | 0.938958 |
| 31. | 6. | 0. | −0.553396 | −0.830921 | −0.775186 |
| 32. | 8. | 0. | −4.440777 | −0.207438 | −0.242581 |
| 33. | 6. | 0. | −2.690607 | −2.428105 | 0.707525 |
| 34. | 8. | 0. | −2.526921 | −3.275283 | −0.159703 |
| 35. | 6. | 0. | −4.035977 | −2.092327 | 1.304477 |
| 36. | 6. | 0. | −4.985067 | −1.455796 | 0.267912 |
| 37. | 6. | 0. | −5.254214 | −2.321310 | −0.988516 |
| 38. | 6. | 0. | −6.565585 | −1.930004 | −1.658096 |
| 39. | 8. | 0. | −5.320632 | −3.698819 | −0.656546 |
| 40. | 6. | 0. | −2.159824 | 1.576992 | −1.981135 |
| 41. | 8. | 0. | −1.773638 | 1.060090 | 0.243223 |
| 42. | 6. | 0. | −2.411665 | 4.619991 | −0.945784 |
| 43. | 1. | 0. | 5.778283 | −4.292754 | −0.804214 |

| | | | | | |
|---|---|---|---|---|---|
| 44. | 1. | 0. | 6.514774 | −3.367097 | 1.392350 |
| 45. | 1. | 0. | 6.224196 | −0.951819 | 1.890205 |
| 46. | 1. | 0. | 4.460025 | −0.372635 | −1.980999 |
| 47. | 1. | 0. | 5.717830 | 1.260141 | 1.129112 |
| 48. | 1. | 0. | 4.341961 | −2.260966 | −3.282728 |
| 49. | 1. | 0. | 3.516152 | 2.364848 | 0.725610 |
| 50. | 1. | 0. | 2.953839 | 0.952045 | −0.149880 |
| 51. | 1. | 0. | 6.819844 | 2.562730 | −1.997172 |
| 52. | 1. | 0. | 7.302523 | 2.292442 | −0.301878 |
| 53. | 1. | 0. | 7.162095 | 0.910024 | −1.420368 |
| 54. | 1. | 0. | 3.835551 | 0.973552 | 2.787390 |
| 55. | 1. | 0. | 3.308127 | −0.453359 | 1.930618 |
| 56. | 1. | 0. | 1.623436 | 0.444988 | 3.441379 |
| 57. | 1. | 0. | 0.504074 | 2.587887 | 3.191292 |
| 58. | 1. | 0. | 2.243322 | 2.853261 | 3.333744 |
| 59. | 1. | 0. | 1.448207 | 2.993535 | 1.757833 |
| 60. | 1. | 0. | −0.356368 | 0.505717 | 2.130867 |
| 61. | 1. | 0. | 1.614750 | −1.422970 | 0.845336 |
| 62. | 1. | 0. | −0.259355 | −2.742573 | 0.179262 |
| 63. | 1. | 0. | −0.149148 | −1.782445 | 3.336109 |
| 64. | 1. | 0. | 0.683451 | −3.097064 | 2.499141 |
| 65. | 1. | 0. | 1.622602 | −1.826993 | 3.284019 |
| 66. | 1. | 0. | −3.972397 | 2.880107 | 0.747373 |
| 67. | 1. | 0. | −4.590331 | 2.443920 | −0.854092 |
| 68. | 1. | 0. | 0.497709 | 3.169993 | −0.686221 |
| 69. | 1. | 0. | −0.340171 | 3.690859 | −2.153492 |
| 70. | 1. | 0. | 1.317554 | 1.124920 | −2.597379 |
| 71. | 1. | 0. | −0.008694 | 0.182440 | −3.307240 |
| 72. | 1. | 0. | 0.184036 | 1.888024 | −3.717055 |
| 73. | 1. | 0. | −1.104989 | 2.804447 | 1.314438 |
| 74. | 1. | 0. | −1.427207 | −1.130056 | −1.356853 |
| 75. | 1. | 0. | 0.330621 | −1.027166 | −1.390129 |
| 76. | 1. | 0. | −3.927688 | −1.419201 | 2.154005 |
| 77. | 1. | 0. | −4.508409 | −3.021574 | 1.633913 |
| 78. | 1. | 0. | −5.919943 | −1.231404 | 0.789847 |
| 79. | 1. | 0. | −4.424237 | −2.136794 | −1.685122 |
| 80. | 1. | 0. | −6.579859 | −0.860623 | −1.888420 |
| 81. | 1. | 0. | −7.408188 | −2.158458 | −0.997214 |
| 82. | 1. | 0. | −6.697832 | −2.492375 | −2.585775 |
| 83. | 1. | 0. | −4.396528 | −3.985030 | −0.554597 |
| 84. | 1. | 0. | −2.586427 | 0.598527 | −2.213145 |
| 85. | 1. | 0. | −2.459566 | 2.288636 | −2.756301 |
| 86. | 1. | 0. | −3.293587 | 4.841267 | −0.339050 |
| 87. | 1. | 0. | −2.717467 | 4.505541 | −1.989631 |
| 88. | 1. | 0. | −1.740241 | 5.481554 | −0.881579 |

**Table S1.4.3.** DP4+ evaluation of theoretical and experimental data for isomer 1 and isomer 2.

| **Funtional** | **Solvent** | **Bassis Set** | **Type of Data** | |
|---|---|---|---|---|
| **mPW1PW91** | **PCM** | **6–311+G(d, p)** | **Unscaled Shifts** | |
| | | **DP4+** | **0.00%** | **100.00%** |
| **Nuclei** | **Sp2** | **Experimental** | **Isomer 1** | **Isomer 2** |
| C | x[1] | 172.1 | 177.2 | 176.7 |
| C | | 34.8 | 35.2 | 36.8 |
| C | | 85 | 86.0 | 87.6 |
| C | | 79.2 | 79.8 | 79.5 |
| C | | 47.1 | 46.8 | 42.0 |
| C | | 48.7 | 51.7 | 50.6 |

| | | | | |
|---|---|---|---|---|
| C | | 105.5 | 103.7 | 105.8 |
| C | | 105.5 | 28.1 | 33.5 |
| C | | 105.5 | 78.2 | 75.5 |
| C | | 105.5 | 36.3 | 36.2 |
| C | | 105.5 | 69.8 | 72.4 |
| C | | 105.5 | 36.79 | 36.03 |
| C | | 105.5 | 28.46 | 28.99 |
| C | | 105.5 | 36.81 | 32.75 |
| C | | 105.5 | 81.37 | 81.76 |
| C | x | 105.5 | 144.66 | 144.98 |
| C | x | 105.5 | 112.55 | 111.75 |
| C | x | 105.5 | 159.05 | 158.46 |
| C | x | 105.5 | 115.38 | 114.87 |
| C | x | 105.5 | 129.65 | 129.53 |
| C | x | 105.5 | 115.07 | 117.43 |
| C | | 105.5 | 13.81 | 13.52 |
| C | | 105.5 | 13.81 | 12.06 |
| C | | 105.5 | 45.30 | 44.55 |
| C | | 105.5 | 13.57 | 15.38 |
| C | | 105.5 | 21.18 | 22.55 |
| C | x | 105.5 | 170.25 | 174.64 |
| C | | 105.5 | 39.66 | 34.64 |
| C | | 105.5 | 81.56 | 76.67 |
| C | | 105.5 | 70.73 | 69.22 |
| C | | 105.5 | 14.75 | 18.39 |
| C | | 105.5 | 54.25 | 54.43 |
| H | | 105.5 | 2.79 | 2.834 |
| H | | 105.5 | 2.499 | 2.343 |
| H | | 105.5 | 1.827 | 2.07 |
| H | | 105.5 | 1.567 | 1.523 |
| H | | 105.5 | 2.353 | 1.792 |
| H | | 105.5 | 1.343 | 1.336 |
| H | | 105.5 | 4.17 | 4.449 |
| H | | 105.5 | 1.334 | 1.338 |
| H | | 105.5 | 3.542 | 3.609 |
| H | | 105.5 | 1.334 | 1.244 |
| H | | 105.5 | 1.334 | 1.244 |
| H | | 105.5 | 1.362 | 1.307 |
| H | | 105.5 | 1.815 | 2.07 |
| H | | 105.5 | 1.815 | 1.599 |
| H | | 105.5 | 4.17 | 4.016 |
| H | x | 105.5 | 6.345 | 6.272 |
| H | x | 105.5 | 6.467 | 6.286 |
| H | x | 105.5 | 6.868 | 6.831 |
| H | x | 105.5 | 6.541 | 6.601 |
| H | | 105.5 | 0.777 | 0.785 |
| H | | 105.5 | 0.631 | 0.499 |
| H | | 105.5 | 2.026 | 2.245 |
| H | | 105.5 | 2.026 | 1.244 |
| H | | 105.5 | 0.929 | 0.828 |
| H | | 105.5 | 1.087 | 1.244 |
| H | | 105.5 | 2.253 | 3.34 |
| H | | 105.5 | 2.026 | 2.244 |
| H | | 105.5 | 5.081 | 4.51 |
| H | | 105.5 | 3.642 | 3.729 |
| H | | 105.5 | 0.986 | 1.134 |
| H | | 105.5 | 2.911 | 3.034 |

[1] x: The atoms are Sp2 hybrid.

| Functional | Solvent? | Basis Set | Type of Data |
|---|---|---|---|
| mPW1PW91 | PCM | 6-311+G(d,p) | Unscaled Shifts |

**2.**

| | Isomer 1 | Isomer 2 | Isomer 3 | Isomer 4 | Isomer 5 | Isomer 6 |
|---|---|---|---|---|---|---|
| sDP4+ (H data) | 0.00% | 100.00% | – | – | – | – |
| sDP4+ (C data) | 0.00% | 100.00% | – | – | – | – |
| sDP4+ (all data) | 0.00% | 100.00% | – | – | – | – |
| uDP4+ (H data) | 0.62% | 99.38% | – | – | – | – |
| uDP4+ (C data) | 19.31% | 80.69% | – | – | – | – |
| uDP4+ (all data) | 0.15% | 99.85% | – | – | – | – |
| DP4+ (H data) | 0.00% | 100.00% | – | – | – | – |
| DP4+ (C data) | 0.00% | 100.00% | – | – | – | – |
| DP4+ (all data) | 0.00% | 100.00% | – | – | – | – |

## Spectra Data of 1 and 2

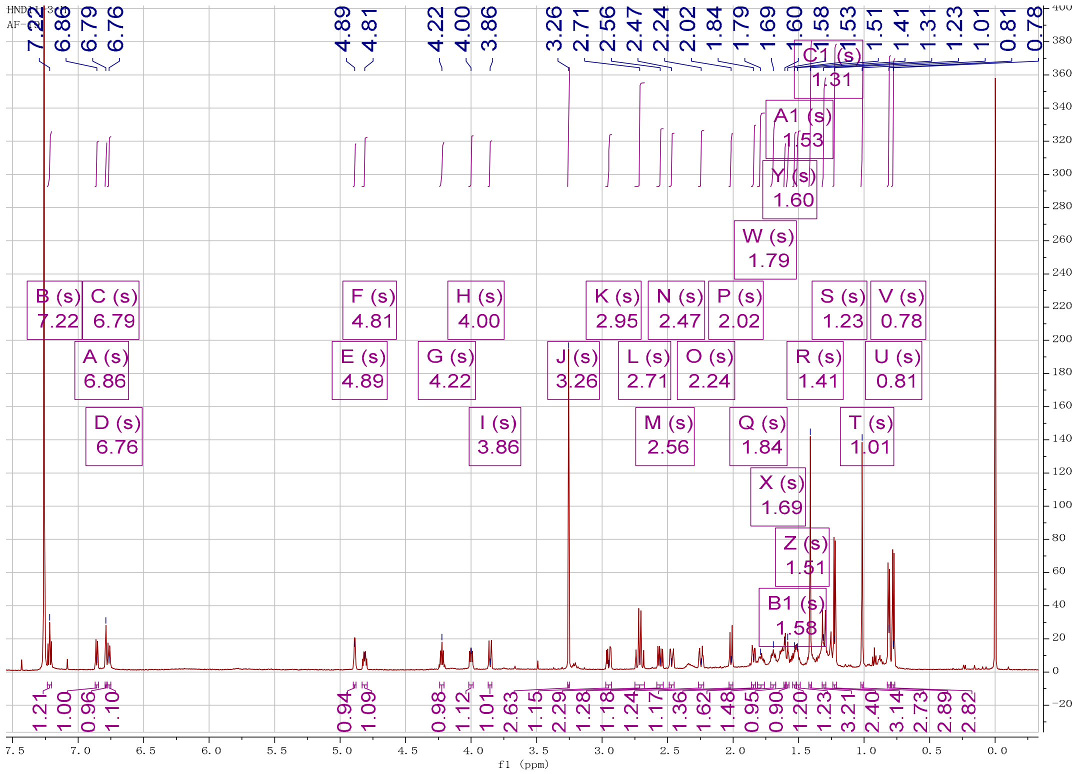


**Figure S2.1.** $^{1}$H NMR spectrum of compound **1** in $CDCl_3$.

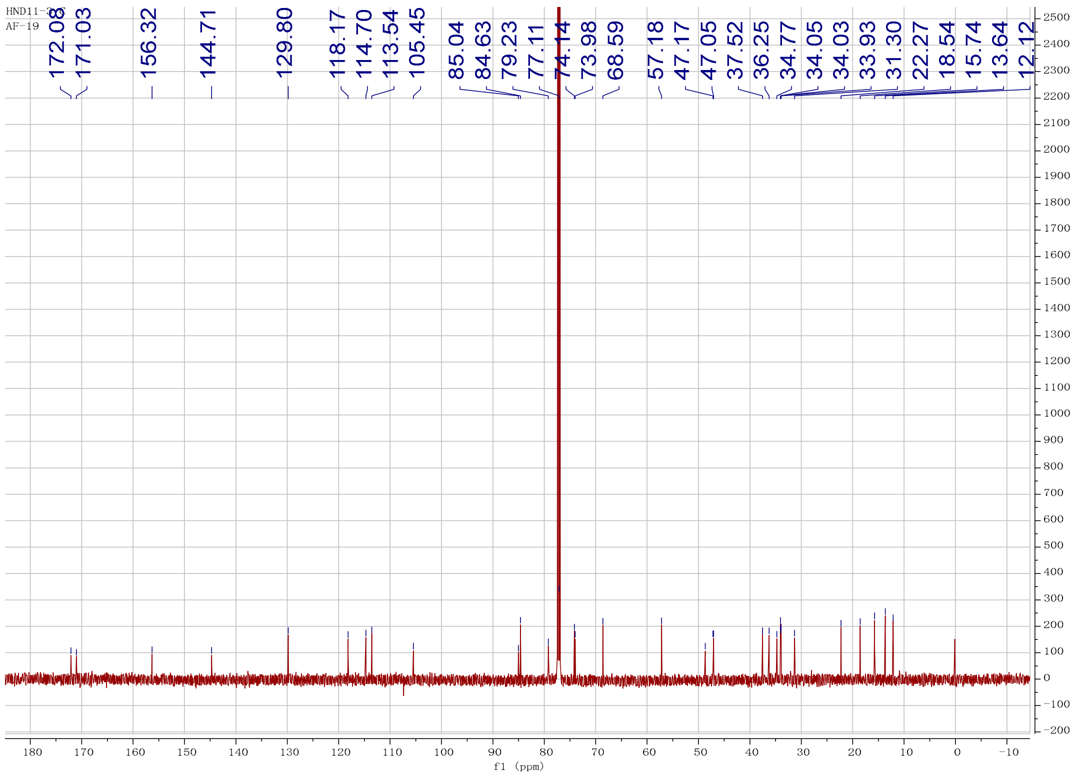

**Figure S2.2.** $^{13}$C NMR spectrum of compound **1** in $CDCl_3$.

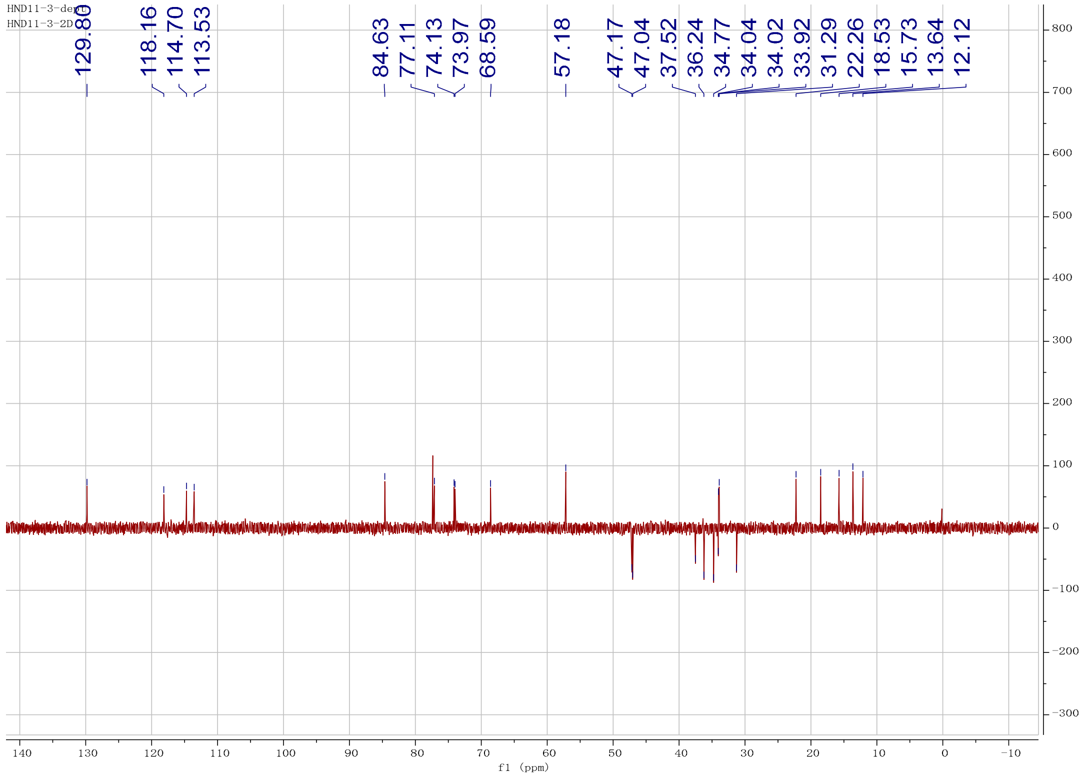

**Figure S2.3.** DEPT spectrum of compound **1** in $CDCl_3$.

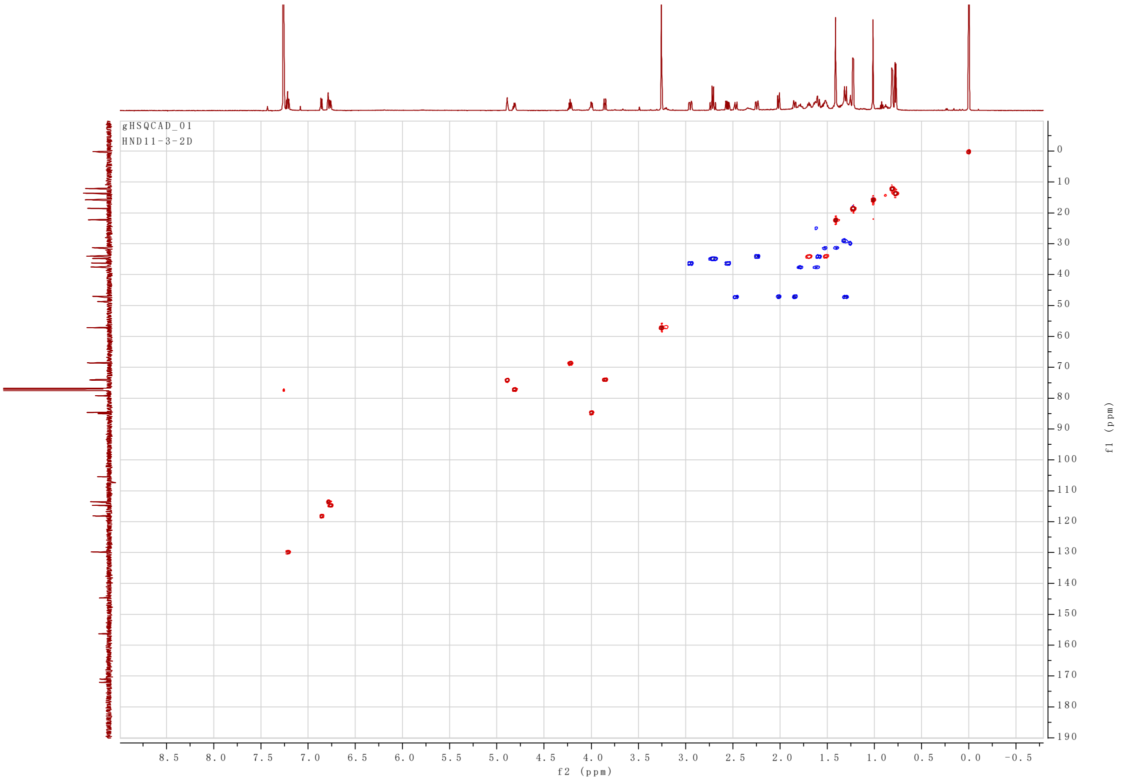


**Figure S2.4.** HSQC spectrum of compound **1** in CDCl3.

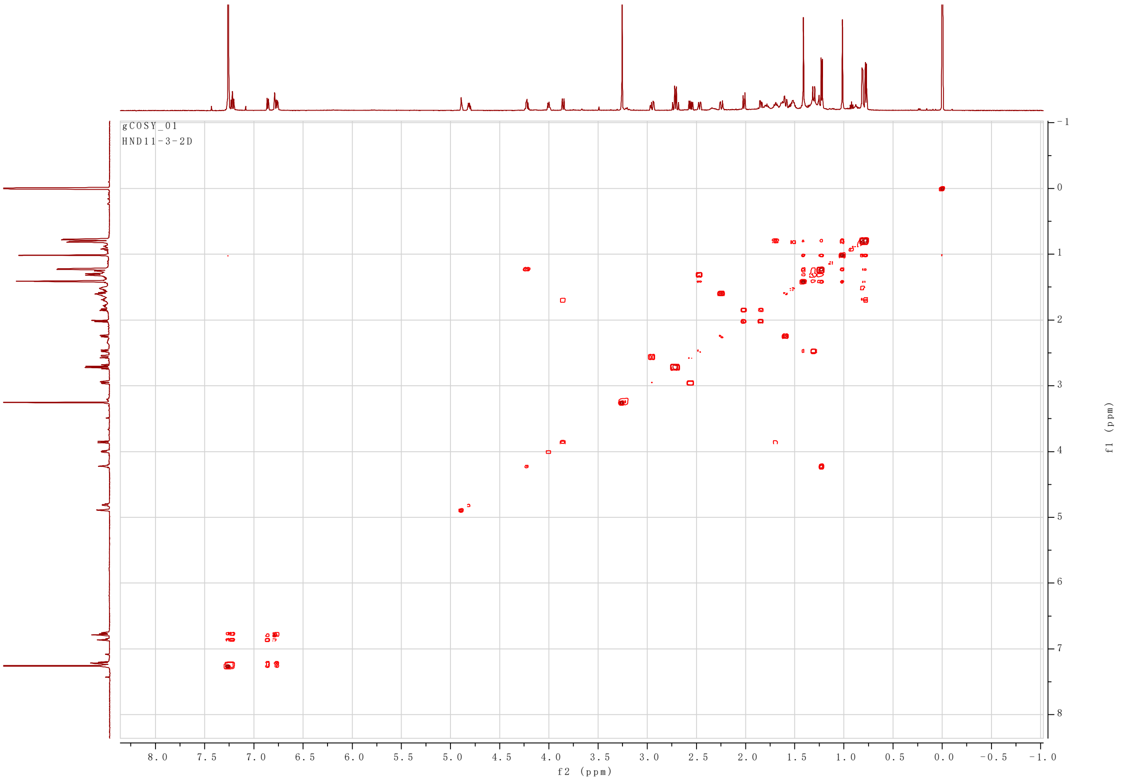


**Figure S2.5.** 1H-1H COSY spectrum of compound **1** in CDCl3.

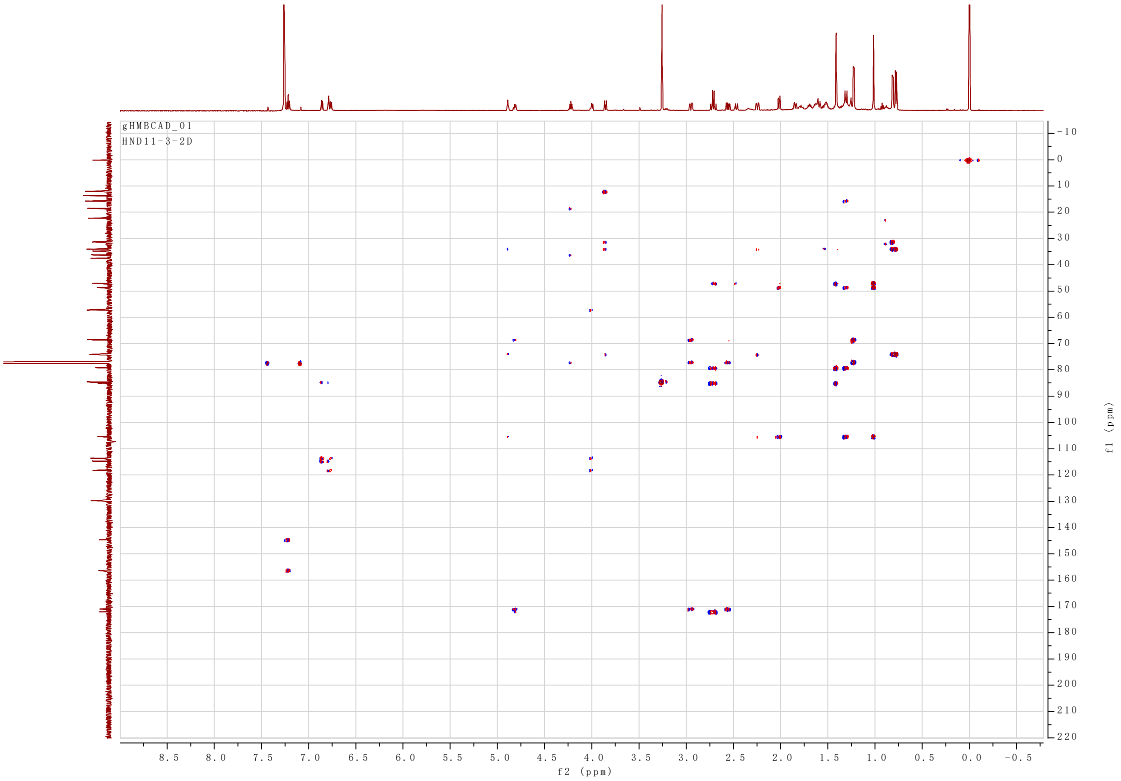


**Figure S2.6.** HMBC spectrum of compound **1** in CDCl3.

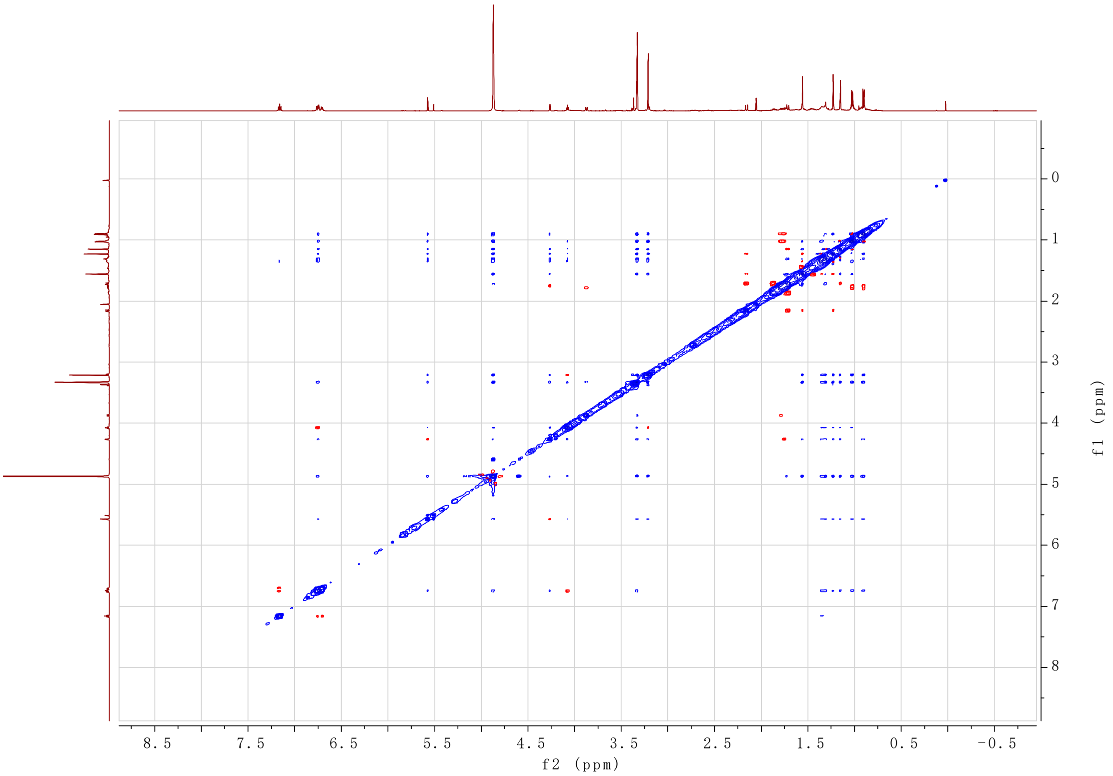


**Figure S2.7.** NOESY spectrum of compound **1** in $CDCl_3$.

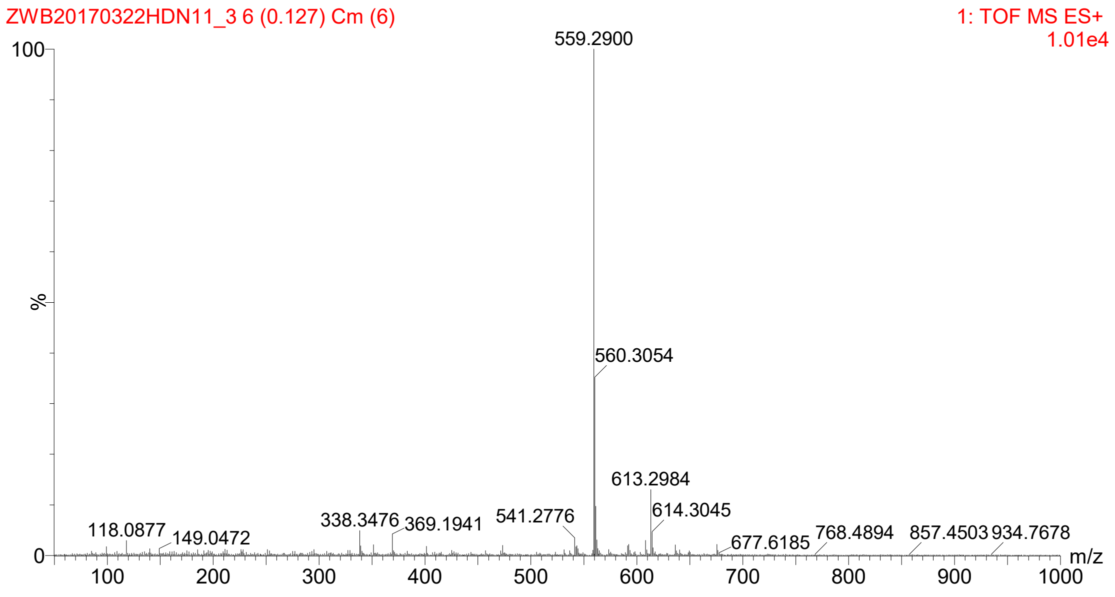


**Figure S2.8.** HRESIMS spectrum of compound **1** in MeOH.

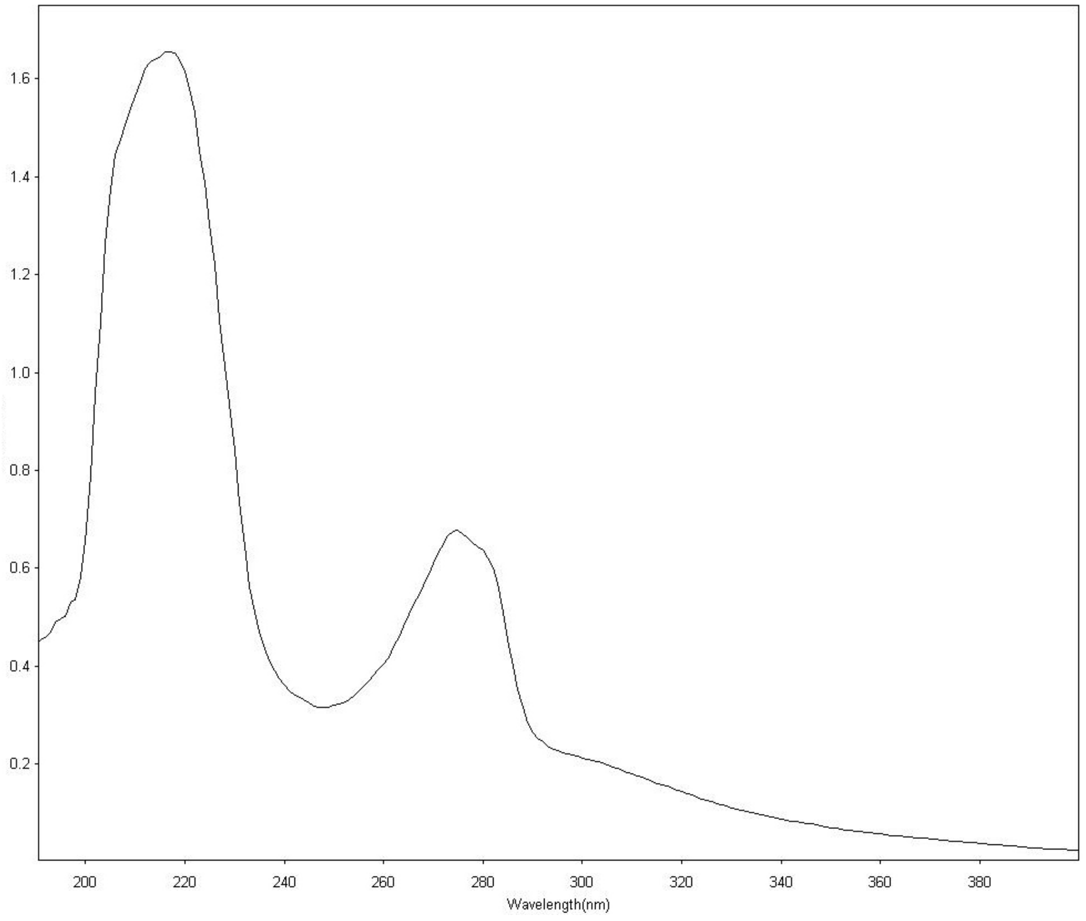


**Figure S2.9.** UV spectrum of Neo-debromoaplysiatoxin G (**1**) in MeOH.

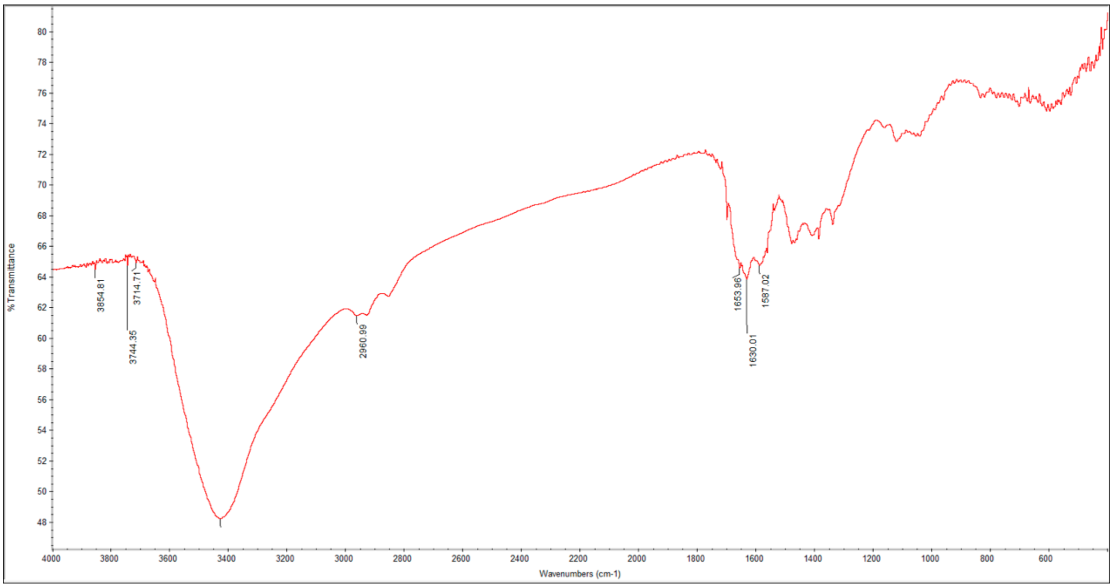

**Figure S2.10.** IR spectrum of Neo-debromoaplysiatoxin G (**1**).

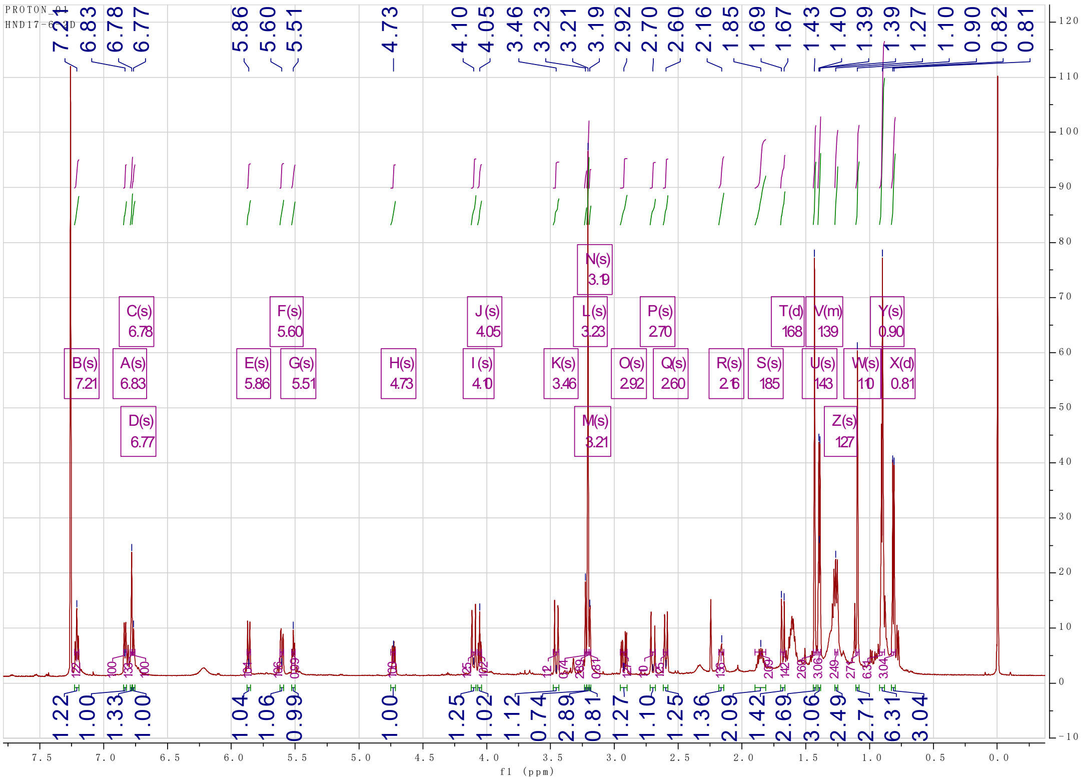

**Figure S2.11.** $^{1}$H NMR spectrum of compound **2** in CDCl3.

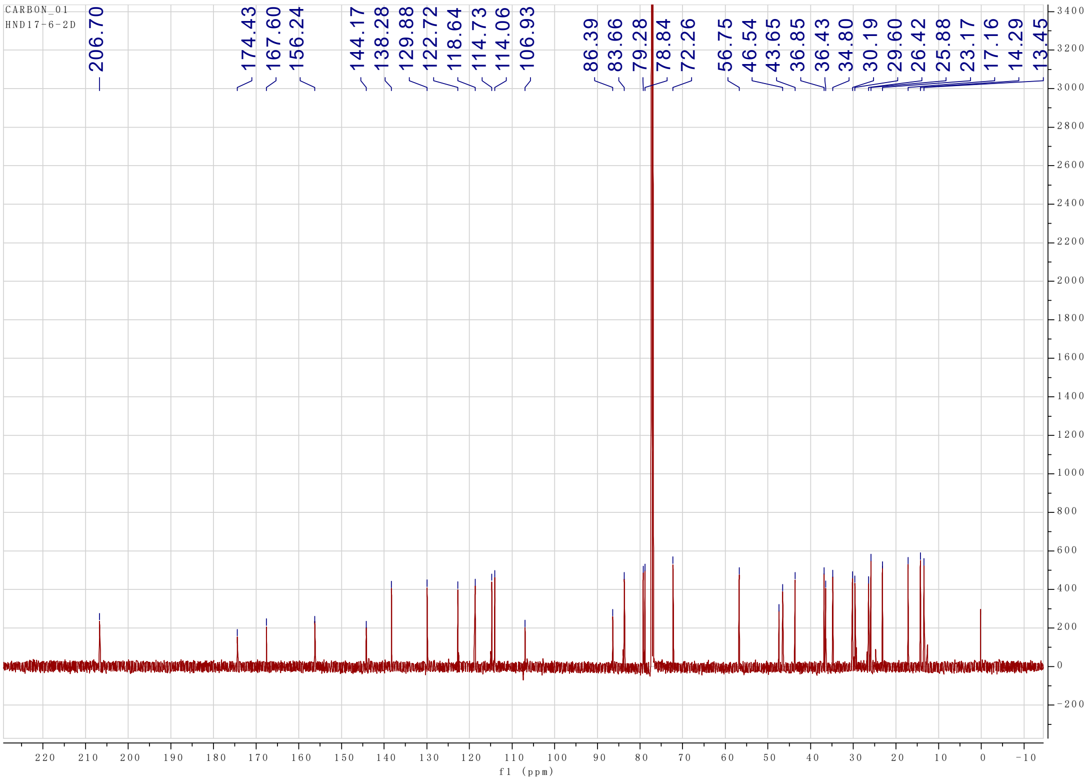


**Figure S2.12.** $^{13}$C NMR spectrum of compound **2** in CDCl3.

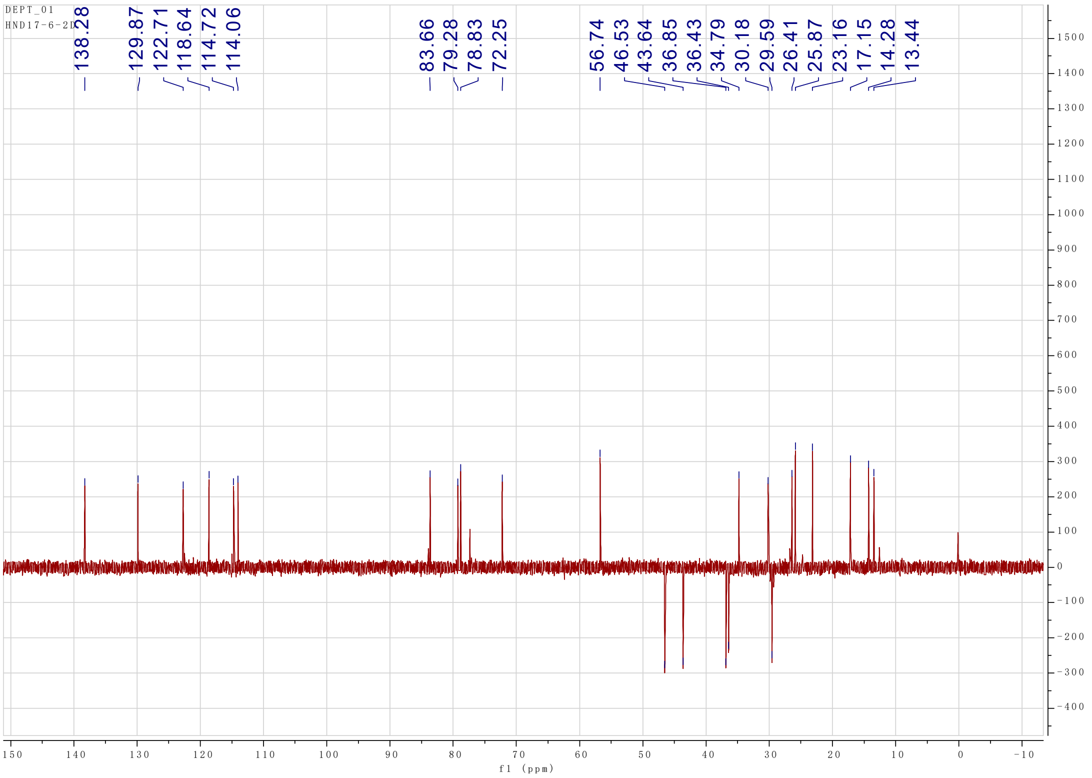


**Figure S2.13.** DEPT spectrum of compound **2** in $CDCl_3$.

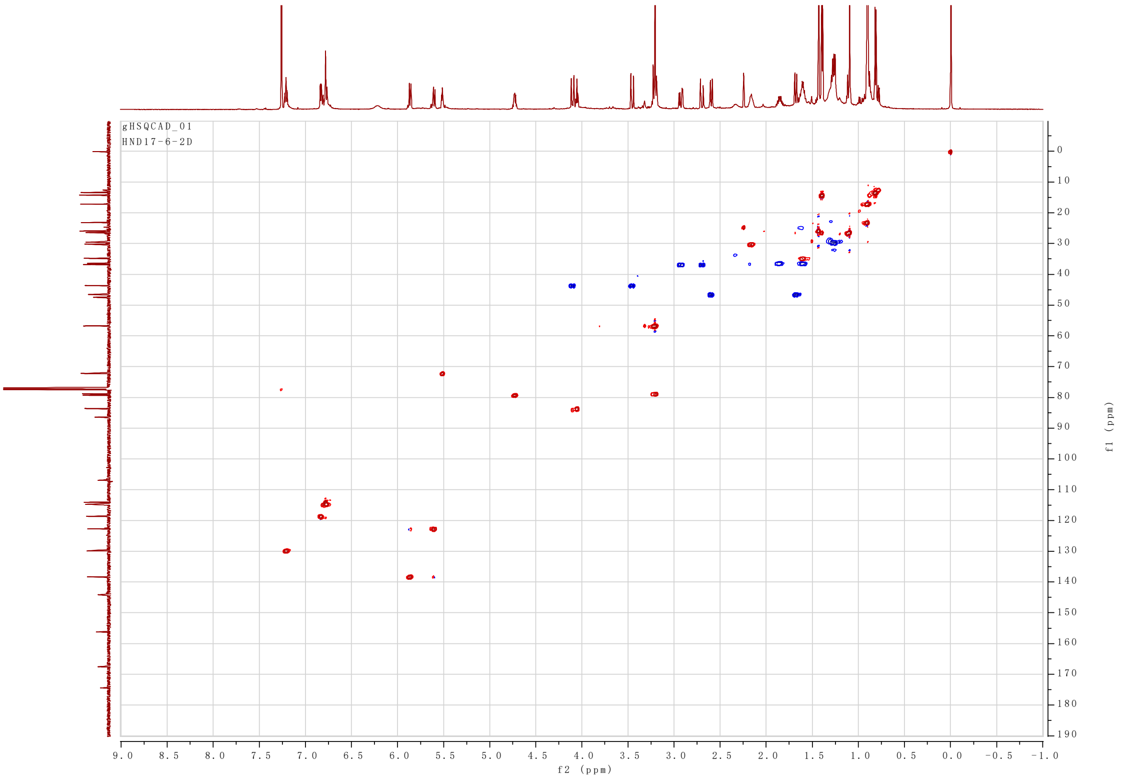


**Figure S2.14.** HSQC spectrum of compound **2** in $CDCl_3$.

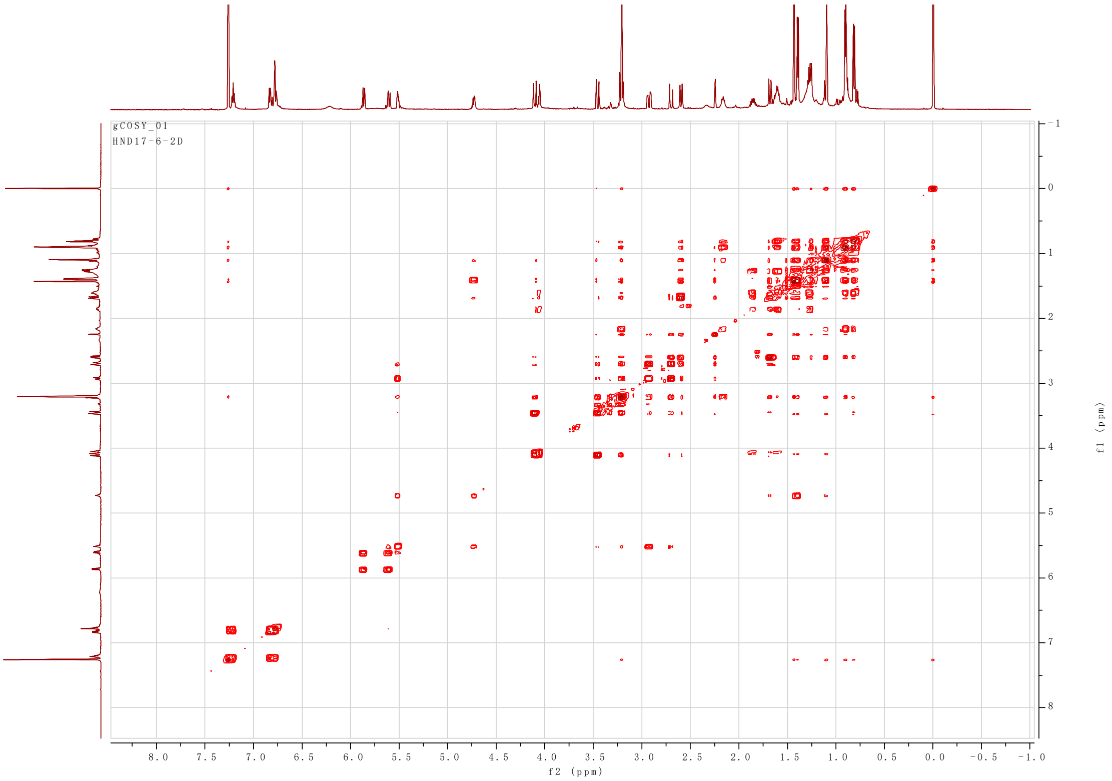


**Figure S2.15.** $^1H$-$^1H$ COSY spectrum of compound **2** in $CDCl_3$.

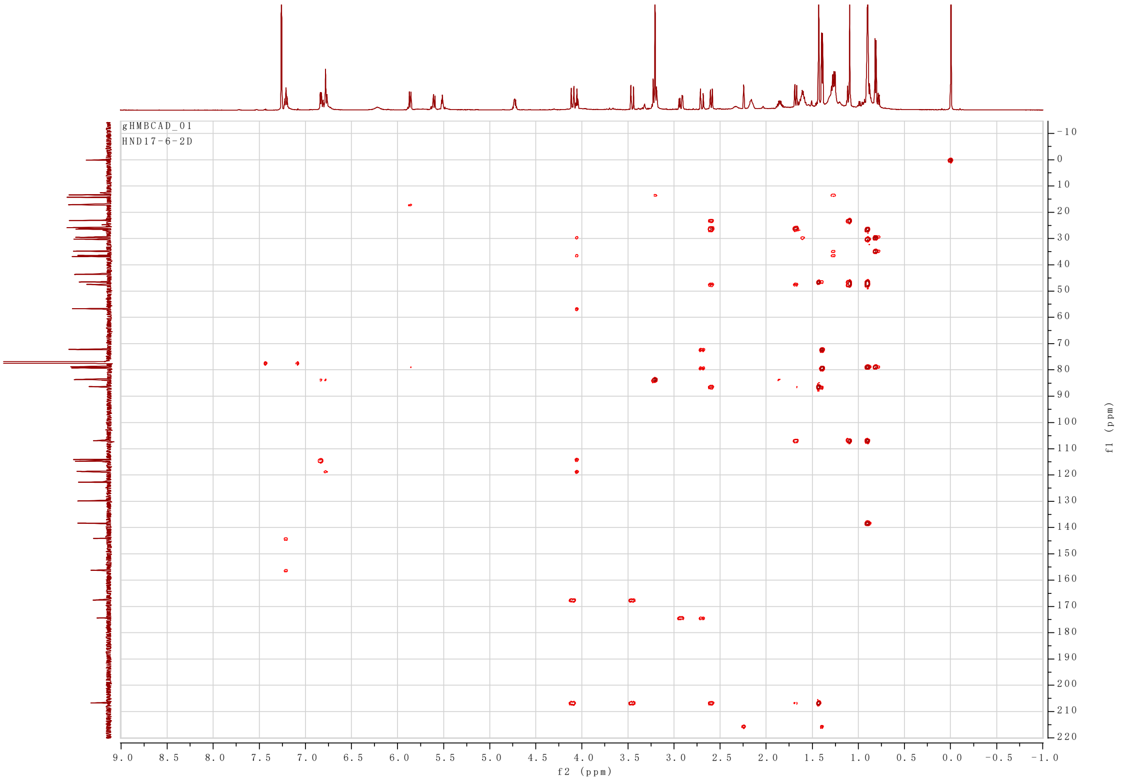


**Figure S2.16.** HMBC spectrum of compound **2** in $CDCl_3$.

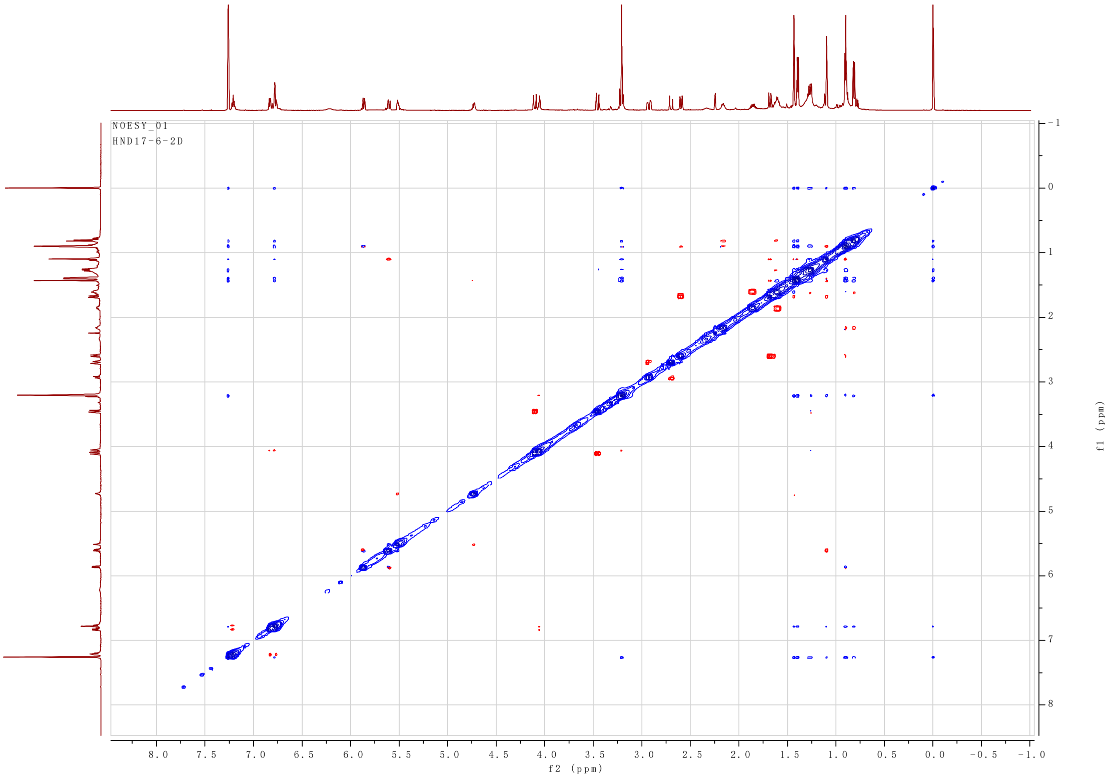


**Figure S2.17.** NOESY spectrum of compound **2** in $CDCl_3$.

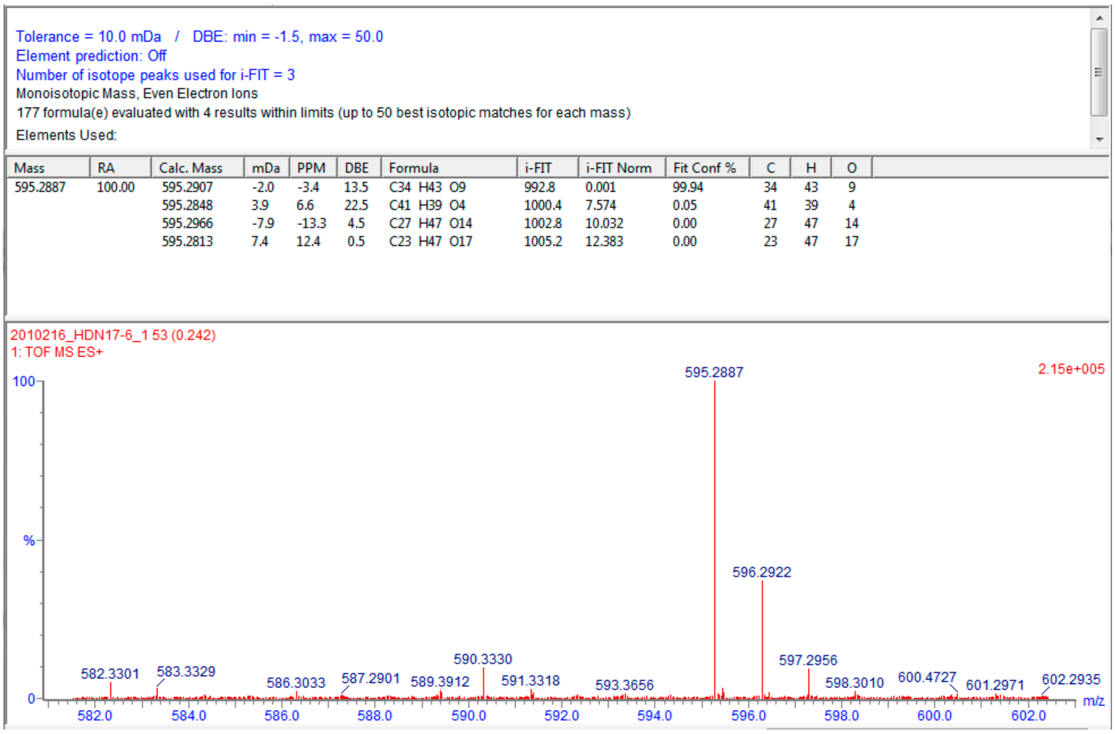


**Figure S2.18.** HRESIMS spectrum of compound **2** in MeOH.

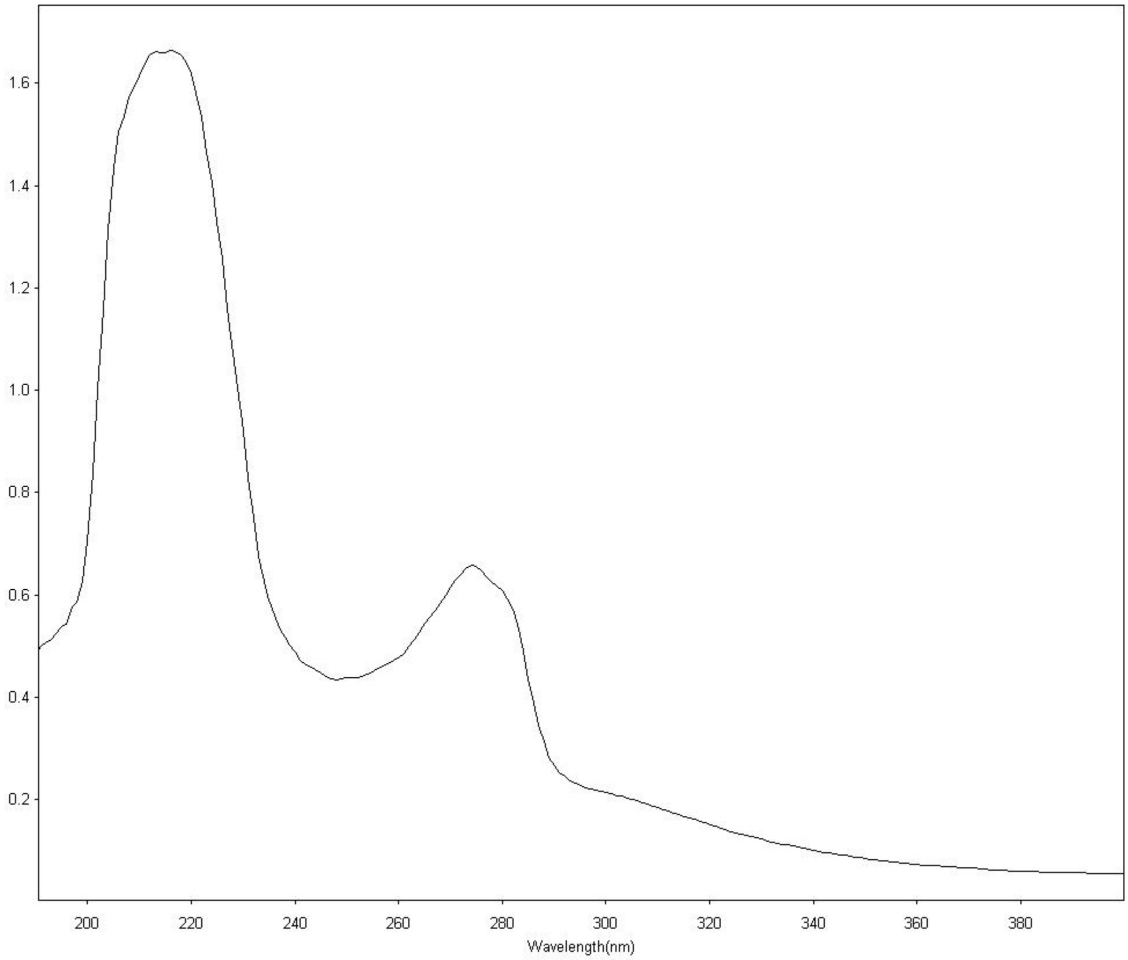


**Figure S2.19.** UV spectrum of Neo-ebromoaplysiatoxin H (**2**) in MeOH.

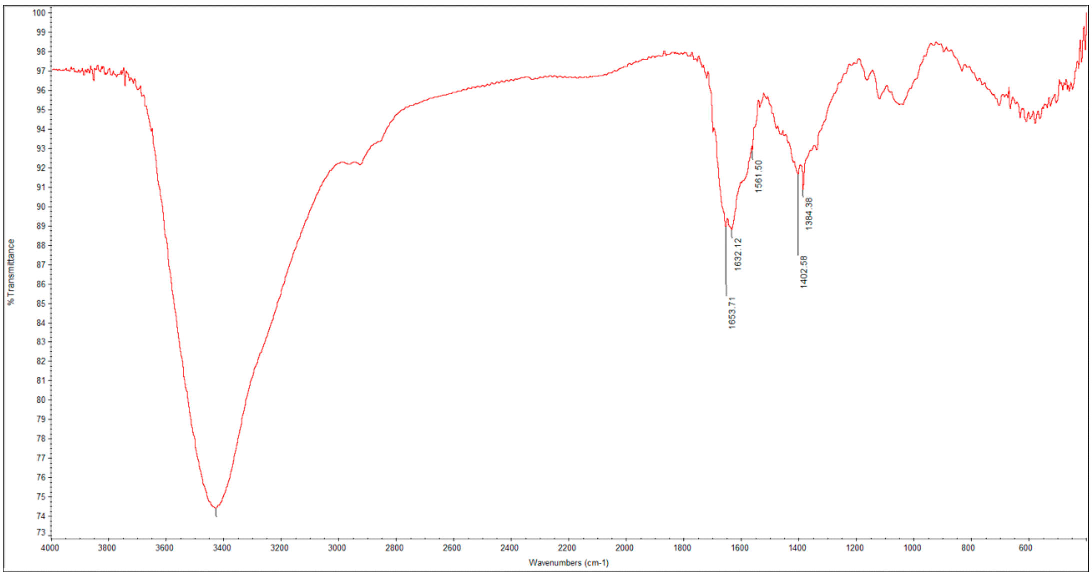


**Figure S2.20.** IR spectrum of Neo-debromoaplysiatoxin H (**2**).

## 3. The Distribution of All ATXs Produced in Different Sea Areas

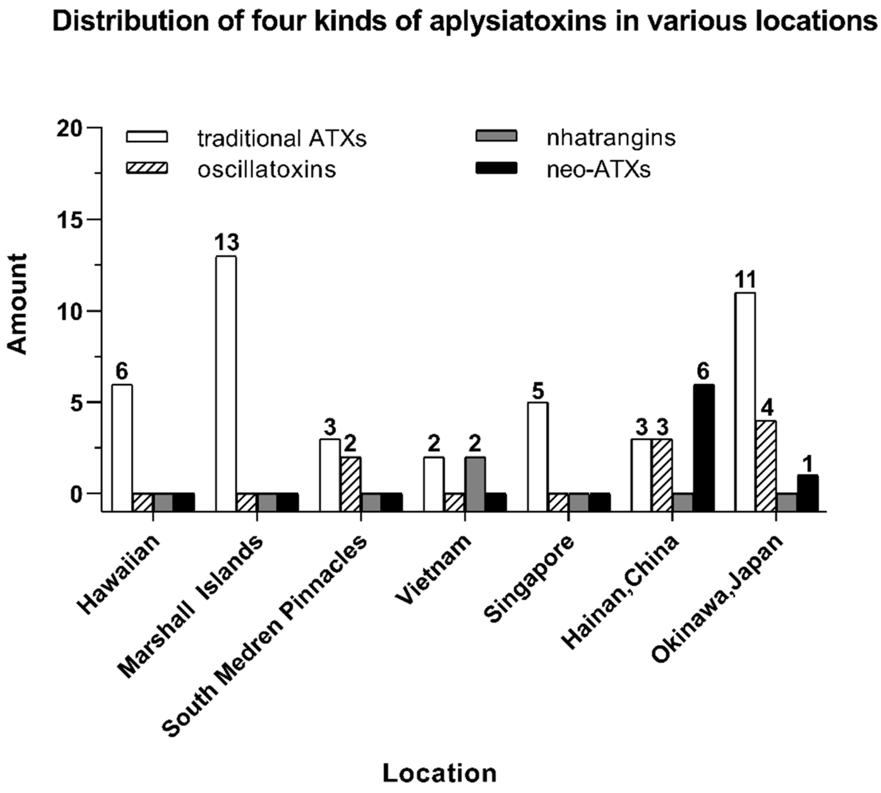


**Figure S3.1.** The distribution of four types of ATXs in various locations (traditional ATXs; oscillatoxins; nhatrangins; intriguing neo-ATXs). The values on the bar represent the number of ATXs of the corresponding type for different sea areas (Table S3.1).

**Table S3.1.** The distribution of all ATXs produced in different sea areas.

| Name | Time | Location | Source | References |
|---|---|---|---|---|
| Aplysiatoxin (1)<br>Debromoaplysiatoxin (2) | 1969 | Hawaiian | *Aplysia pulmonica*<br>*Dolabella auricularia*<br>*Dolabrfera dolabrfera*<br>*Stylocheilus longicauda* | 1 |
| | 1974 | Hawaiian | *Stylocheilus longicauda* | 2 |
| | 1980 | Hawaiian windward Oahu | *Lyngbya majuscula* | 3 |
| Debromoaplysiatoxin (2) | 1977 | Enewetak Atoll, Marshall Islands | *Oscillatoria* sp. | 4 |
| Oscillatoxin A (3)<br>17-bromooscillatoxin A(4)<br>17,19-dibromooscillatoxin A (5)<br>19-bromoaplysiatoxin(6)<br>19-bromodebromoaplysiatoxin(7)<br>21-bromooscillatoxin (8)<br>19,21-dibromoaplysiatoxin(9)<br>Anhydroaplysiatoxins(10)(11)(12)(13)<br>Anhydrooscillatoxin A(14) | 1978 | Enewetak Atoll, Marshall Islands | *Oscillatoria nigroviridis*<br>*Schizothrix calcicola* | 5 |
| Debromoaplysiatoxin(2)<br>OscillatoxinA(3) | 1984 | Enewetak Atoll, Marshall Islands | *Lyngbya majuscula* | 6 |
| Oscillatoxin B1 (15)<br>Oscillatoxin B2 (16)<br>31-noroscillatoxin B(17)<br>Oscillatoxin D (18)<br>30-methyloscillatoxin D(19) | 1985 | South Medren Pinnacles | *Lyngbya majuscula*<br>*Oscillatoria nigroviridis*<br>*Schizothrix calcicola* | 7 |
| Aplysiatoxin (1)<br>Debromoaplysiatoxin (2) | 1996 | Waiehu, Maui, Hawaiian | *Gracilaria coronopifolia* | 8 |
| Aplysiatoxin(1)<br>Debromoaplysiatoxin(2)<br>ManauealideA-C(20)(21)(22)<br>Anhydebromoaplysiatoxins(23) | 1998 | Hawaiian | *Gracilaria coronopifolia* | 9 |
| Anhydroaplysiatoxin(10)<br>Anhydebromoaplysiatoxins(23)<br>NhatranginA(24)<br>NhatranginB(25) | 2010 | Vietnam | *Lyngbya Majuscula* | 10 |
| Aplysiatoxin(1)<br>Debromoaplysiatoxin(2)<br>Ahydebromoaplysiatoxins(23)<br>3-methoxyaplysiatoxin(26)<br>3-methoxydebromoaplysiatoxin(27) | 2014 | Pulau Seringat Kias, Singapore | *Trichodesmium erythraeum* | 11 |
| Neo-debromoaplysiatoxinA(28)<br>Neo-debromoaplysiatoxinB(29) | 2018 | Sanya, Hainan, China | *Lyngbya* sp. | 12 |
| Neo-debromoaplysiatoxinC(30)<br>Neo-debromoaplysiatoxinD(31)<br>OscillatoxinE(32)<br>OscillatoxinF(33)<br>Debromoaplysiatoxin(2)<br>30-methyloscillatoxinD(19)<br>Anhydebromoaplysiatoxins(23)<br>3-methoxydebromoaplysiatoxin(27) | 2019 | Sanya, Hainan, China | *Lyngbya* sp. | 13/14 |
| Aplysiatoxin(1)<br>Debromoaplysiatoxin(2)<br>Anhydroaplysiatoxins(10)<br>OscillatoxinB1(15)<br>OscillatoxinB2(16)<br>Anhydebromoaplysiatoxins(23)<br>2-hydroxyanhydroaplysiatoxin(34)<br>17-BromooscillatoxinB2(35)<br>17-Bromo-4-hydroperoxyoscillatoxinB2(36)<br>4-hydroperoxyoscillatoxinB2(37)<br>17-Bromo-4,26-epoxyoscillatoxinB2(38) | 2019 | Okinawa, Japan | *Moorea producens* | 15 |
| OscillatoxinI(39)<br>OscillatoxinG(40)<br>OscillatoxinH(41)<br>17-Bromo-30-methyloscillatoxinD(42) | 2019 | Okinawa, Japan | *Moorea producens* | 16 |
| Neo-debromoaplysiatoxinE(43)<br>Neo-debromoaplysiatoxinF(44) | 2019 | Sanya, Hainan, China | *Lyngbya* sp. | 17 |
| Neo-aplysiatoxinA(45) | 2020 | Okinawa, Japan | *Moorea producens* | 18 |

**Table S3.2.** NMR Data for debromoaplysiatoxin in CDCL3 (δ in ppm, *J* in *Hz*).

| Pos. | Debromoaplysiatoxin | |
|---|---|---|
| | **$\delta_H$ (*J* in Hz)** | **$\delta_C$** |
| 1 | | 169.2 |
| 2 | a 2.76, d (12.7)<br>b 2.52, d (12.7) | 46.9 |
| 3 | | 98.8 |
| 4 | 1.84, m | 35.7 |
| 5 | a 1.62, t (13.1)<br>b 1.05, dd (13.1, 3.6) | 41.1 |
| 6 | | 39.0 |
| 7 | | 100.08 |
| 8 | a 2.68, dd (14.7, 3.0)<br>b 1.71, dd (14.8, 3.6) | 33.6 |
| 9 | 5.23, m | 73.2 |
| 10 | 1.71, m | 35.4 |
| 11 | 3.93, dd (10.9, 2.1) | 69.8 |
| 12 | 1.52, m | 34.2 |
| 13 | a 1.31, m<br>b 1.39, m | 31.2 |
| 14 | a 1.97, m<br>b 1.63, m | 36.1 |
| 15 | 4.0, dd (8.5, 6.5) | 85.8 |
| 16 | | 145.9 |
| 17 | 6.92, t (1.1) | 114.6 |
| 18 | | 158.3 |
| 19 | 6.71, dt (7.9, 1,1) | 115.0 |
| 20 | 7.13, t (7.8, 7.8) | 129.8 |
| 21 | 6.84, dt (7.9, 1,1) | 119.3 |
| 22 | 0.79, d (6.8) | 13.6 |
| 23 | 0.71, d (6.9) | 13.0 |
| 24 | 0.83, s | 26.8 |
| 25 | 0.80, s | 16.5 |
| 26 | 0.86, d (6.8) | 23.6 |
| 27 | | 170.4 |
| 28 | a 2.92, dd (18.2, 11.1)<br>b 2.87, dd (18.1, 2.8) | 34.6 |
| 29 | 5.23, m | 74.3 |
| 30 | 4.03, m | 67.0 |
| 31 | 1.12, d (6.4) | 17.7 |
| 15−$OCH_3$ | 3.17, s | 56.6 |

## References


1. Watson M. Aspects of the pharmacology, chemistry, and biology of the midgut gland toxins of some Hawaiian sea hares, especially Dolabella auricularia and Aplysia pulmonica [D]. 1969.
2. Kato Y, Scheuer P J. ChemInform Abstract:Aplysiatoxin and debromoaplysiatoxin, constituents of the marine mollusk stylocheilus longicauda (quoy and gaimard, 1824) [J]. Chemischer Informationsdienst, 1974, 5(24): 2245−2246.
3. Serdula M, Bartolini G, Moore R E, et al. Seaweed itch on windward Oahu [J]. Hawaii Medical Journal, 1982, 41(7): 200−201.

4. Mynderse J S, Moore R E, Kashiwagi M, et al. Antileukemia activity in the Oscillatoriaceae: isolation of debromoaplysiatoxin from Lyngbya [J]. Science,1977, 196(4289): 538−540.
5. Mynderse J S, Moore R E. Toxins from blue−green algae: structures of oscillatoxin A and three related bromine−containing toxins [J]. The Journal of Organic Chemistry, 1978, 43(11): 2301−2303.
6. Moore R E, Blackman A J, Cheuk C E, et al. Cheminform Abstract: Absolute stereochemistries of the aplysiatoxins and oscillatoxin A [J]. Cheminform, 1984, 15(49): 2484−2489.
7. Entzeroth M, Blackman A J, Mynderse J S, et al. Structures and stereochemistries of oscillatoxin B, 31−noroscillatoxin B, oscillatoxin D, and 30−methyloscillatoxin D [J]. The Journal of Organic Chemistry, 1985, 50(8): 1255−1259.
8. Nagai H, Yasumoto T, Hokama Y. Aplysiatoxin and debromoaplysiatoxin as the causative agents of a red alga Gracilaria coronopifolia poisoning in Hawaii [J]. Toxicon, 1996, 34(7): 753−761.
9. Nagai H, Kan Y, Fujita T, et al. Manauealide C and Anhydrodebromoaplysiatoxin, Toxic Constituents of the Hawaiian Red Alga, Gracilaria coronopifolia [J]. Bioscience, Biotechnology, and Biochemistry, 1998, 62(5): 1011−1013.
10. Chlipala G E, Tri P H, Nguyen V H, et al. Nhatrangins A and B, aplysiatoxin− related metabolites from the marine cyanobacterium Lyngbya majuscula from Vietnam [J]. Journal of Natural Products, 2010, 73(4): 784−787.
11. Gupta D K, Kaur P, Leong S T, et al. Anti−Chikungunya Viral Activities of Aplysiatoxin−Related Compounds from the Marine Cyanobacterium Trichodesmium erythraeum [J]. Marine Drugs, 2014, 12(1): 115−127.
12. Han B−N, Liang T−T, Keen L, et al. Two Marine Cyanobacterial Aplysiatoxin Polyketides, Neo−debromoaplysiatoxin A and B, with K+ Channel Inhibition Activity [J]. Organic Letters, 2018, 20(3): 578−581.
13. Tang Y−H, Wu J, Fan T−T, et al. Chemical and biological study of aplysiatoxin derivatives showing inhibition of potassium channel Kv1.5 [J]. RSC Advances, 2019, 9(14): 7594−7600.
14. Tang Y−H, Liang T−T, Fan T−T, et al. Neo−debromoaplysiatoxin C, with new structural rearrangement, derived from debromoaplysiatoxin [J]. Natural Product Research, 2019, 5: 1−6.
15. Nagai H, Watanabe M, Sato S, et al. New aplysiatoxin derivatives from the Okinawan cyanobacterium Moorea producens [J]. Tetrahedron, 2019, 75(17): 2486−2494.
16. Nagai H, Sato S, Iida K, et al. Oscillatoxin I: A New Aplysiatoxin Derivative, from a Marine Cyanobacterium [J]. Toxins, 2019, 11(6): 366−372.
17. Fan T−T, Zhang H−H, Tang Y−H, et al. Two New Neo−debromoaplysiatoxins−A Pair of Stereoisomers Exhibiting Potent Kv1.5 Ion Channel Inhibition Activities. Marine drugs 2019, 17(12), 652−664.
18. Kawaguchi M, Satake M, Zhang B−T, et al. Neo−Aplysiatoxin A Isolated from Okinawan Cyanobacterium Moorea Producens [J]. Molecules, 2020, 25(3): 457−464.
